# Uneven recombination rate and linkage disequilibrium across a reference SNP map for common bean (*Phaseolus vulgaris* L.)

**DOI:** 10.1371/journal.pone.0189597

**Published:** 2018-03-09

**Authors:** Matthew W. Blair, Andrés J. Cortés, Andrew D. Farmer, Wei Huang, Daniel Ambachew, R. Varma Penmetsa, Noelia Carrasquilla-Garcia, Teshale Assefa, Steven B. Cannon

**Affiliations:** 1 Department of Agricultural & Environmental Science, Tennessee State University (TSU), Nashville, Tennessee, United States of America; 2 Colombian Corporation for Agricultural Research (CORPOICA), C.I. La Selva, Rionegro, Department of Antioquia, Colombia; 3 National Center for Genome Resources (NCGR), Santa Fe, New Mexico, United States of America; 4 Iowa State University (ISU), Ames, Iowa, United States of America; 5 University of California, Davis (US-D), California, United States of America; 6 United States Department of Agriculture - Agricultural Research Service (USDA-ARS), Corn Insects and Crop Genetics Research Unit, Ames, Iowa, United States of America; Julius Kühn-Institut, GERMANY

## Abstract

Recombination (R) rate and linkage disequilibrium (LD) analyses are the basis for plant breeding. These vary by breeding system, by generation of inbreeding or outcrossing and by region in the chromosome. Common bean (*Phaseolus vulgaris* L.) is a favored food legume with a small sequenced genome (514 Mb) and n = 11 chromosomes. The goal of this study was to describe R and LD in the common bean genome using a 768-marker array of single nucleotide polymorphisms (SNP) based on Trans-legume Orthologous Group (TOG) genes along with an advanced-generation Recombinant Inbred Line reference mapping population (BAT93 x Jalo EEP558) and an internationally available diversity panel. A whole genome genetic map was created that covered all eleven linkage groups (LG). The LGs were linked to the physical map by sequence data of the TOGs compared to each chromosome sequence of common bean. The genetic map length in total was smaller than for previous maps reflecting the precision of allele calling and mapping with SNP technology as well as the use of gene-based markers. A total of 91.4% of TOG markers had singleton hits with annotated Pv genes and all mapped outside of regions of resistance gene clusters. LD levels were found to be stronger within the Mesoamerican genepool and decay more rapidly within the Andean genepool. The recombination rate across the genome was 2.13 cM / Mb but R was found to be highly repressed around centromeres and frequent outside peri-centromeric regions. These results have important implications for association and genetic mapping or crop improvement in common bean.

## Introduction

Common bean is an important food legume with interesting genetics that is also a good protein and micronutrient source for many consumers around the world [1]. The crop has two major cultivated genepools derived from the Andes mountains of South America (Andean genepool) and from the entire corridor of Mesoamerica through Central America from what is today Mexico to Colombia (Mesoamerican genepool). The half-way split in population structure between two genepools of cultivated common beans is well-documented by multiple molecular marker studies [2,3]. The relative diversity in each cultivar genepool is subject to different estimates, with some studies showing equal diversity [4] or greater diversity in one or the other genepool [5–9].

Linkage disequilibrium (LD) analysis is related to population genetics and has been the basis for genetic and association mapping done in common beans and other inbreeding crops, especially with advanced generation populations [10]. LD is the non-random association of alleles between different loci and can be based on population structure, physical proximity or epistatic interactions.

LD is influenced, among other factors, by the rate of chromosomal recombination (R) within a species across multiple generations of inbreeding or cross-breeding and depends on the mating system of the plant and the location within the genome [11]. Thus, regional LD values vary with different parts of the genome depending on physical constraints, such as chromosomal structure, location of repetitive DNA segments or ribosomal DNA and other cytogenetic factors that impede recombination.

Apart from these issues, epistatic interactions may create non-random associations among unlinked loci, and genomic differentiation between subspecies can limit LD decay [12]. Since R varies, certain regions of chromosomes can also have higher or lower LD due to reduced or increased recombination fraction; and this in turn can be related to epistasis, linkage drag or gene flow. Variability in R can be due to chromosomal context and structure, so that greater crossing-over occurs in euchromatin segments in gene-dense regions, compared to heterochromatin, gene-poor regions or centromeres [13,14]. Furthermore, recombination is generally higher within tandemly repeated gene families such as resistance gene analog loci, because of concerted evolution.

Single nucleotide polymorphism (SNP) markers have been developed over the past six years for common beans. The first SNPs to be developed for the crop were designed based on amplicon re-sequencing of mapping parents [15,16] consensus legume gene sequences and [17] and gene alignments [18]. These were then used for diversity assessment but with small germplasm panels [15,17]. Further non-gene based SNP markers were then made in larger numbers by different research groups than our own but predominantly for genetic mapping [19–21]. Genotyping-by-sequencing (GBS) and re-sequencing are other sources of SNPs in common bean that provide dense maps in inter-genepool crosses [22] but which do not validate the SNPs as mappable markers as was done for Illumina-based SNP markers [20]. In general, SNP markers are abundant in plant genomes and useful for R and LD evaluations by mapping or genetic diversity analysis.

LD analysis and evaluations of R can be organized based on a single chromosomal region, various genomic regions or the entire genome, especially when working with SNP markers. For example, SNP markers have been useful in locus specific studies [2,23–26] or across the genome for single-genepool diversity panels [19,27]. Meanwhile, the Illumina SNPs from Blair *et al*. [17] were shown to be effective in distinguishing between genepools and saturating inter-genepool common bean maps, as they are based on highly conserved trans-legume orthologous sequences called TOGs as explained by Lee *et al*. [28]. However, to date few genetic maps, with the exception of those of Bhakta *et al*. [22] or Song *et al*. [20], have a predominance of SNP markers and none are based on TOG type markers useful for study of LD. Bhakta *et al*. [22] used a Recombinant Inbred Line (RIL) population to locate recombination hotspots and regions of suppressed recombination but did not associate this with LD estimates. In terms of SNP mapping, Song *et al*. [20] used an F2 mapping population and the RIL population mentioned in Schmutz et al. [5] has not been widely used.

The goals of this study were 1) to provide the genetic and physical map locations for the gene-based SNP assays developed by Blair et al. [17] which are a basic set of Trans-legume Orthologous Group (TOG) markers in common bean; 2) to compare the new maps with whole genome sequence of Schmutz et al. [5], which is the most complete sequence to date for the species; 3) to estimate R from mapping in the BAT93 x Jalo EEP558 reference population which is the most widely-available and long-standing core RIL set [29] available to the bean community; and 4) to estimate LD across the entire genome based on the diversity for SNP marker alleles in Andean and Mesoamerican publically-accessible diversity panels. As a complement to all of these studies, we have incorporated the genetic information in the Legume Information System.

## Materials and methods

### Plant material and DNA extraction

The plant material used in this study were 1) a recombinant inbred lines (RIL) of the inter-genepool, Andean x Mesoamerican genetic mapping population BAT93 x Jalo EEP558, described as a core mapping population by Freyre *et al*. [29] and used in the comparative genetic mapping of SSR markers by Blair *et al*. [30] and the addition of other SSR markers by Grisi *et al*. [31] and 2) a LD germplasm panel of 186 genotypes that included 71 Andean and 115 Mesoamerican genotypes known as the validation set from Blair *et al*. [17]. These were diverse common beans with known cultivar race assignments [32] that represented the full diversity of common beans randomly selected from the reference collection described in Blair *et al*. [4]. The plants for the DNA extraction of the LD panel and the mapping population were grown in a greenhouse in trays with four rows per tray each with five seed per row, representing each RIL genotype. The newly-emerged first true leaves and shoot tips of the plants in a row were harvested for the five plants and used in DNA extractions following the method described in Afanador *et al*. [33], which is a modification of the CTAB method. Briefly, 2 g of fresh tissue was ground in liquid N_2_ to a fine powder, which was mixed with extraction buffer and incubated at 65 C in a 15 mL Falcon tube. Protein removal was accomplished using two chloroform–isoamyl alchohol extractions at 1:1 ratio, which were shaken with the tissue homogenate and centrifuged at 10,000 rpm, removing the upper aqueous layer for DNA precipitation. The resulting purified DNA was quantified on a Hoefer DyNA Quant 2000 fluorometer and diluted to a standard concentration (200 ng/μl) for use in SNP marker evaluation.

### SNP marker evaluation and genetic mapping

The SNP markers used for this study were from the legume trans-legume orthologous gene (TOG) series made for common bean from the sequencing of BAT93 and Jalo EEP558 parental genotypes as described in Blair *et al*. [17]. The markers were assayed by an Illumina GoldenGate (GG) chip array with 768 locus specific features that were known to be polymorphic for the BAT93 x Jalo EEP558 population based on sequencing of amplicons from the two parental genotypes (Cook laboratory, UC-Davis). Aliquots of 10 μl of the standard 200 ng/μl DNA concentration for the genotypes were sent to the UC-Davis Genome Center DNA Technologies facility for the assay according to standard protocols for GG chip evaluation (http://dnatech.genomecenter.ucdavis.edu/). SNP genotyping calls were made with Bead-studio software package (Illumina, San Diego, CA, USA).

Alleles for the population were then used for genetic mapping carried out in Mapdisto v. 2.0 [34] assuming an RIL model and using the “create groups” command both with and without anchor markers. Anchor markers including restriction fragment length polymorphism (RFLP), amplified fragment length polymorphism (AFLP), random amplified polymorphism (RAPD) markers from Freyre *et al*. [29] and simple sequence repeat (SSR) markers from Blair *et al*. [30] were placed on linkage groups with the “place marker” command which helped to identify the correct chromosome of the common bean genome and associate known marker positions with each linkage group. Kosambi function was used to estimate centiMorgan (cM) genetic distances from the recombination fraction and drawing scale was 1. Heterozygous SNP calls were considered missing data for the sake of genotyping but very few were found in the RIL population (≤0.5%) due to the advanced generation (F_11_).

### Physical mapping and comparisons with the genetic map

The genetic map from the methodology described above was predominantly made of TOG markers and was aligned with the physical map through sequence comparisons through a nucleotide BLASTn (Basic Local Alignment Search Tool) (blast.ncbi.nlm.nih.gov) search using default parameters for significance. The query sequence included the 120 bp of flanking sequences, or 60 bp on both left and right sides, of each of the common bean SNP markers compared to the chromosomal sequences available for *Phaseolus vulgaris* [5]. These pseudo-molecules were from the version 1.0 genome sequences for common bean at the Phytozome website (phytozome.jgi.doe.gov). The most homologous physical position of each SNP amplicon sequence was estimated based on the lowest E-value hit found by the similarity search and recorded in Mega base pairs (Mb). Multiple matches were not considered for the TOG markers. The physical positions of the SNP markers were used in the construction of a customized comparative map for all 11 chromosomes carried out with the software R (v2.15.1 from R Core Team, code available from senior authors) showing physical (Mb) and genetic (cM) distances. Chromosomal identity and the orientation per chromosome were based on the physical map. After collecting mapping information on both scales, scatter plots were created with R software to analyze the relationship between linkage map distance (y-axis, cM) and physical distance (x-axis, Mb) for each chromosome. Polynomial line-fitting was used to determine the points of inflection and flattening in the curves fit to each of the chromosomal plots; with these indicating suppressed recombination typical of the centromeres and peri-centromeric (pCENR) regions as described in Bhakta *et al*. [22]. The centromeres according to Schmutz *et al*. [5] were marked as circles with the extent of centromeric repeats from the circles shown as outlying bars.

### Gene comparisons

As a core set of SNPs, the highly conserved markers from this study are useful because of their association with orthologous loci across Expressed Sequence Tags from the transcriptomes of *Medicago truncatula*, *Lotus japonicus* and *Glycine max*. It was for this reason that the markers were named TOG (trans-legume orthologous group) markers, which are similar to the COS (conserved orthologous sequence) markers that have been useful in Solanaceous plant species and advocated by Lee *et al*. [28]. To further study the nature of the TOG markers we searched the flanking sequences of the SNPs against genes predicted in common bean. Gene matches with the COS markers were determined by identifying overlaps in genomic sequence coordinates between the top placement for each marker, and the gene models in the *Phaseolus vulgaris* G19833 genome assembly, Phytozome v1.0. The overlap (intersection) between markers and genes was calculated using the bedtools “intersect” function [35].

### LD analysis

Genepools based on population structure were determined with non *a priori* criteria of stratification using STRUCTURE 2.3.2 [36], a burn-in length of 50,000 iterations and a run length of 100,000 iterations with five replicates as described in Blair *et al*. [17]. Polymorphic information content (PIC), genome-wide levels of genetic diversity between any pair of accessions (π) (Nei 1987) and minor-allele-frequency (MAF) for each SNP marker and for each of the genepools was then calculated with the program DnaSP 5.10 [37). These observed distributions were compared with the expectations of the Wright–Fisher neutral model of molecular evolution using coalescent simulations with 5,000 repetitions.

The overall LD was estimated by calculating the square value of correlation coefficient (r^2^) between all pairs of markers with the software package TASSEL 2.1 [38]. D′ was also calculated. Although D′ and r^2^ capture different aspects of the gametic associations [39], they were highly correlated and only the latter is used here for comparative purposes. Only marker loci with minor allele frequency values above 0.05 and having at least 80% successful calls among the sample set were included further for LD analyses. P-values for each r^2^ estimate were obtained with a two-sided Fisher’s exact test as done in the same program.

The LD values between all pairs of marker loci on a single linkage group are shown as triangle LD plots. Meanwhile, TASSEL was used to estimate the general view of genome-wide LD patterns and evaluate ‘block-like’ LD structures. LD plots against genetic and physical map distance were generated by using the estimates of the genetic map, where only r^2^ values with P<0.001 and among markers within the same linkage group were included. A curve was fitted to describe the trend of LD decay using a polynomial regression model implemented in R software.

### Databasing and interactive visualizations

The genotypes used are in S1 Table. The gene matches with the COS markers, and the gene descriptors for these genes, are in S2 Table. The markers and flanking sequences with physical map locations based on blastn, are in S3 Table. The map information can also be interactively visualized in the Legume Information System (“LIS”; https://legumeinfo.org), in several contexts. The TOG-based map is viewable using CMap v. 1.01 [40] with markers placed onto the *Phaseolus vulgaris* v1.0 GBrowse genome viewer, at https://legumeinfo.org/genomes/gbrowse/Pv1.0. Underlying data files are at the LIS database: https://legumeinfo.org/data/public/Phaseolus_vulgaris/mixed.map1.7PMp/.

## Results

### Genetic mapping and physical map comparisons

**Fig 1:**
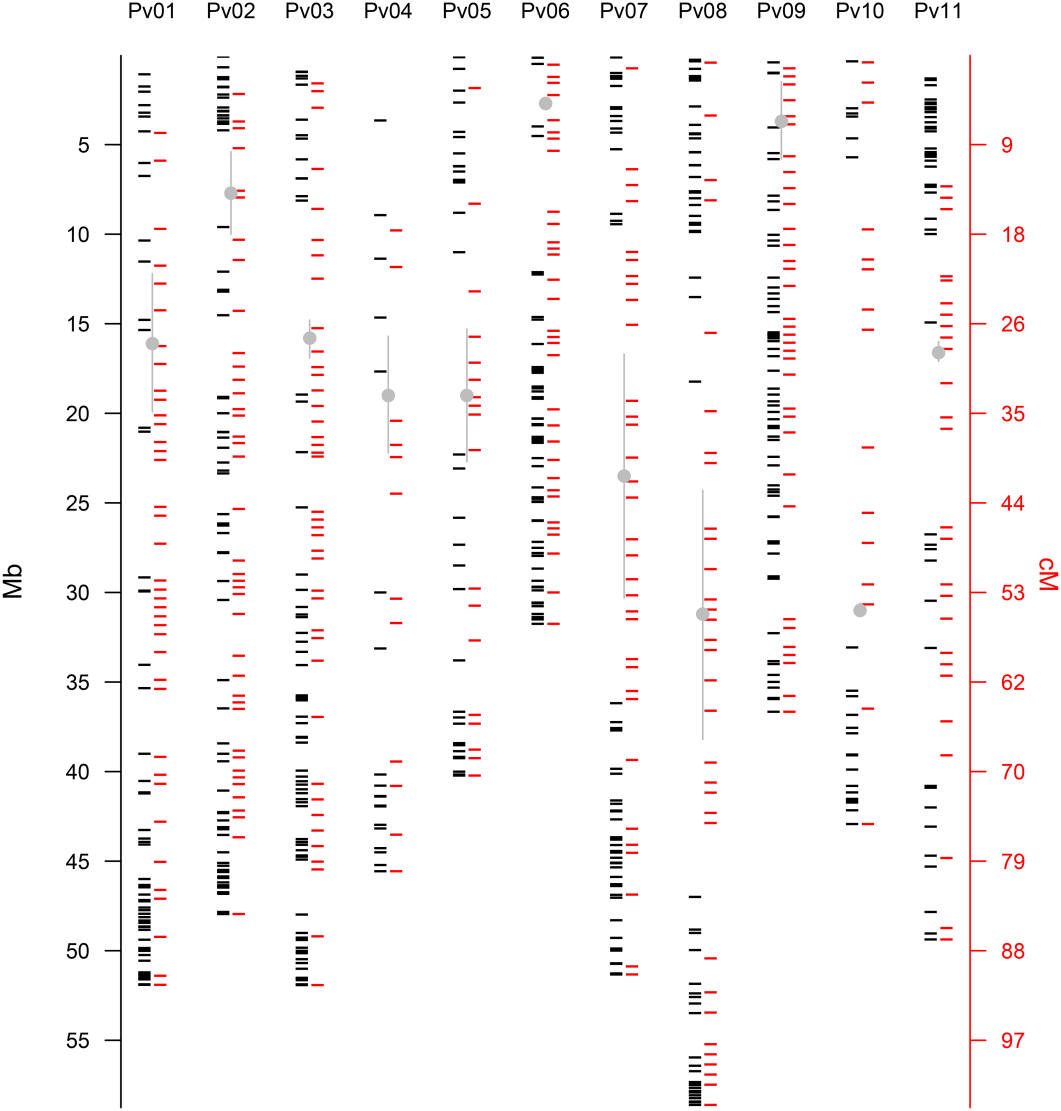
Relationship for each chromosome (labelled as Pv) between linkage map position (on the cM axis) and physical map position (on the Mb axis) for 11 chromosomes of common bean based on the SNP marker mapping described in this text and the BAT93 x JaloEEP558 recombinant inbred line population.

The SNP based BAT93 x JaloEEP558 genetic map was constructed with all the single copy TOG markers from Blair *et al*. [17] and was stored as the Cook lab map in the LIS database. This SNP only map was corrected by adding legacy markers as chromosomal anchors so as to identify each chromosome. In the this step of mapping, SNP markers were added to a total of 265 previously mapped markers, including dominant type markers AFLP and RAPD [29], as well as co-dominant type markers including RFLPs from the Bng and D series and SSRs from the BM and BMd series [30], resulting in a map of nearly 1000 markers. Heterozygosity and low signal were found in 7 and 32 SNP markers, respectively, and these were not included which amounted to a high marker success rate of 94.9% as expected given the parental source of the TOGs being the same as the parents of the mapping population, namely BAT93 and Jalo EEP558. The full distance of the map was 1762.5 cM with all but three SNP markers anchored (Table 1) with 1.8 cM between markers. In of the refined genetic mapping, our goal was to determine the relationships between SNP markers and the co-dominant anchor markers of Blair *et al*. [30]. Groups were found with a minimum LOD of 6.0 and organized by “best order” command in Mapdisto. In this map, there were a total of 812 markers and the genetic distance of the total map was reduced to 1097.5 cM (Fig 1, S1 Fig). Linkage groups ranged from 46.5 cM (Pv9) to 147 cM (Pv1) in length. Average length of the linkage group was 99.8 cM with average between-marker distance of 1.35 cM. This map was stored as the Blair lab “mixed” map in the LIS database at https://legumeinfo.org/data/public/Phaseolus_vulgaris/mixed.map1.7PMp/.

**Table 1:** Physical and genetic mapping and map distance in base pairs (bp) or centiMorgans for each linkage group on the G19833 reference genome v 2.1 or in the BAT93 x Jalo EEP558 population, respectively, using SNP markers from the trans-legume orthologous genes (TOG) combined with other types of markers, including restriction fragment length polymorphism (RFLP) markers of the Bng and D series, simple sequence repeat (SSR) markers and isozyme, protein or phenotypic markers.

	Initial Genetic Distance		Refined Genetic Distance						Physical Distance			Comparison
Linkage Group	Total Markers in LG	Final Distance (cM)	TOG SNP markers	RFLP-markers	SSR-markers	Isozyme, Protein Phenotypic	Total high LOD	Final Distance (cM)	Chromosome length (Phytozome)	bp/cM (initial)	bp/cM (refined)	G / P Ratio (cM/Mb)
Pv1	104	230.1	79	7	2	0	88	147.0	52,159,049	226,680	354,823	2.82
Pv2	145	374.5	93	6	3	2 (Chs,I)	104	140.6	49,012,014	130,873	348,592	2.87
Pv3	119	191.1	77	7	0	0	84	114.4	52,266,928	273,506	456,879	2.19
Pv4	37	105.1	22	4	3	1 (Me)	30	84.7	45,799,695	435,773	540,728	1.85
Pv5	62	103.9	40	6	1	2 (Aco2,Diap)	49	82.8	40,676,787	391,499	491,266	2.04
Pv6	101	171.4	78	4	3	0	85	83.7	31,960,678	186,468	381,848	2.62
Pv7	82	104.9	88	6	3	2 (Chi,Phs)	99	108.3	51,729,989	493,136	477,655	2.09
Pv8	93	160.9	84	6	1	0	91	99.2	59,650,056	370,728	601,311	1.66
Pv9	90	72.3	77	4	2	0	83	46.5	37,463,265	518,164	805,662	1.24
Pv10	43	158.6	27	3	2	0	32	72.2	43,227,687	272,558	598,721	1.67
Pv11	89	89.7	61	6	0	0	67	67.5	50,184,061	559,466	743,468	1.35
**TOTAL**	**981**	**1762.5**	**726**	**59**	**20**	**7**	**812**	**1097.5**	**514,130,209**	**291,705**	**468,456**	2.13

Of the 812 genetic markers in the final map, all 768 SNP markers were tested for physical mapping to the common bean genome sequence from Schmutz *et al*. [5]. In total, 66 markers had multiple hits indicating possible paralogous gene sequences and the remaining 702 markers were single copy BLAST hits indicating a single corresponding genome location. Genetic to physical map comparisons (S2 Table) showed that for each linkage group the genetic map size was generally well correlated (r = 0.67, P<0.01) with the physical length of the chromosome. The average genetic to physical map ratio across the genome for this new maps was 2.13 cM / Mb (Table 1) while the number of base pairs (bp) per cM was 291 Kb for the initial map and 468 Kb for the refined map based on high LOD values. Variability in the genetic to physical distance ratio ranged from 1.24 cM / Mb for Pv09 to 2.87 cM / Mb for Pv02.

**Fig 2:**
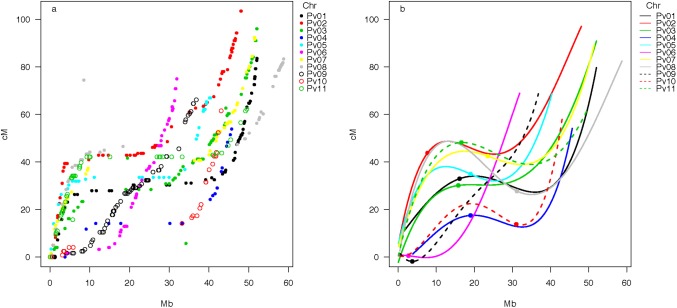
Correlation between linkage map distance (cM) and physical distance (Mb) for the SNP markers across each of the eleven chromosomes of common bean (Pv) showing (a) Absolute values and (b) polynomial fitted lines. Filled dots in (b) mark the centromeres according to Schmutz *et al*. (2014).

**Fig 3:**
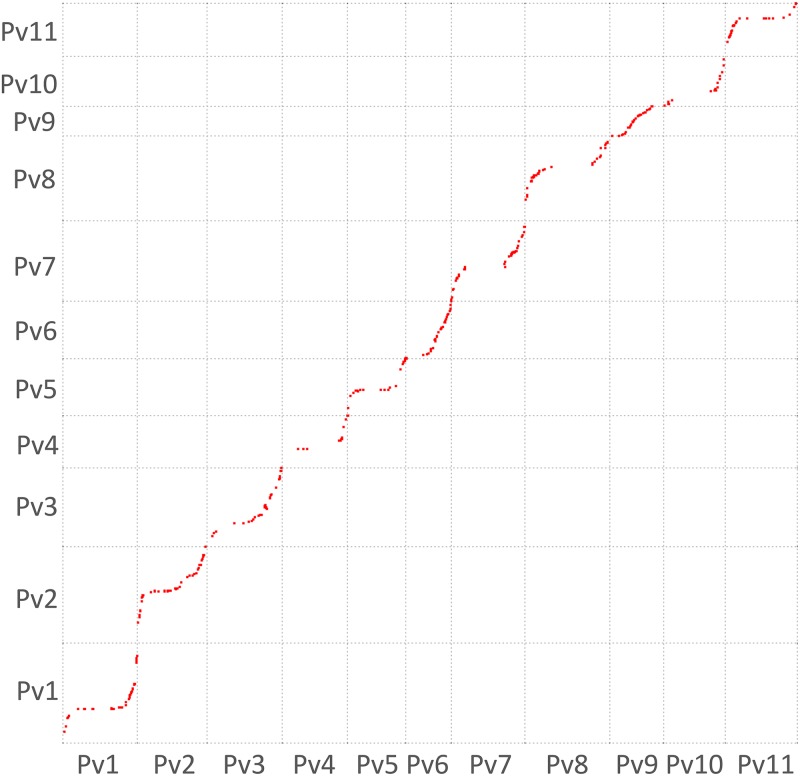
SNP marker amplicon similarity hits in the common bean genome from physical chromosome position (y-axis) to genetic position to (x-axis) with all chromosomes placed end-to-end and oriented per sequence information.

The cM / Mb scatter plots for all common bean chromosomes (Fig 2) and for each chromosome (Fig 3, S2 Fig) agreed with this conclusion that genetic to physical map distances were consistent across the chromosomes. In addition, almost all of the chromosome / linkage group comparisons could be fit with sigmoidal curves, with steep line sections of high recombination, gene-rich regions and flatter plateaus of low recombination. The location of low recombination in the separate or combined scatterplots for each linkage group agreed with the positioning of centromeres and surrounding gene poor regions corresponding to pericentromeric regions (pCENRs) in the physical/genetic map figure. It was notable that linkage groups Pv6 and Pv9 had more even distributions of genes represented by SNP markers, while Pv4, Pv10 and Pv11 had large gaps between TOG markers outside of the pCENR, presumably due to the prevalence of resistance gene clusters (RGCs) on large interstitial segments of the chromosomes corresponding to these linkage groups [5].

### Uniqueness and utility of gene based SNP markers

Of the 768 new TOG markers, the majority, a total of 702 sequences had highly significant singleton hits to predicted or actual genes in the *Phaseolus vulgaris* genome. Meanwhile 66 markers had possible multiple hits under the parameter of difference of e^-10^ from the best hit to next best hit and an E-value threshold of 1e^-30^. At the 1e^-40^ level, 497 TOG markers were exclusive hits to one gene; while in a total number of cases two TOG markers corresponded to the same gene. No difficulties were found searching for the BAT93/Jalo EEP558 derived TOG markers against the genome assembly for G19833, which was the genotype used in the sequencing project of Schmutz *et al*. [5]. It is worth noting that the best e-value (or bit score) achievable for a given query sequence depended to a certain extent on the length of the SNP-flanking sequences, which in almost all cases was 60 bp on both sides of the SNP and 121 bp total. Only one marker, Pv_TOG902802_2_002_1031, had short sequences of 82 bp, which gave a unique hit that was still more significant than 1e^-30^. The results of gene correspondences is given in S3 Table, along with associated gene descriptors, domains, symbols and ontology assignments.

Since the TOG markers were well distributed, it was important to note that some of the new SNPs could be useful in substituting phenotypic or legacy molecular markers. In the case of phenotypically useful markers the virus resistance gene called dominant *I* on linkage group b02d, at the end of the short arm of chromosome Pv02 was flanked at 0.9 cM by three new SNP markers, namely TOG961744_119, TOG906764_834 and TOG906764_376 in this highly recombinogenic and evolutionarily active region that has been amply characterized for the understanding of the necrotic response to BCMNV strains of bean common mosaic virus [41].

Another well-characterized gene with new flanking SNP markers was the locus for phaseolin protein (*Phs*) which influences seed size and where TOG897715_56 and TOG897715_587 were genetically linked at 1.7 cM. Several examples of SNPs linked to isozymes mapped by Freyre et al. [29] were observed for aconitase (*Aco2*), chalcone synthase (*ChS*), chitanase (*Chi*) and diaphorase (*Diap*); while the substitution of RFLP markers by SNPs is self-evident.

### LD patterns within genepools

From Blair *et al*. [17] we knew that the reference germplasm set for the study of LD was heavily structured and had ideal K-value of two, based on 736 SNP markers. Genepool race substructure in that study was weak. Using the information on genepool identity of each genotype, we calculated parameters for polymorphism and LD across the entire collection and within each subpopulation at K = 2. For the first parameter, the distribution of minor-allelic-frequency (MAF) classes was skewed towards high frequencies when computed across genepools, but matched more closely the Wright-Fisher neutral model when computed within genepool (data not shown). This indicated the overall division into two genepools as was previously described [17] but that race structure was not nearly as evident as genepool structure because MAF classes within genepool were not evident. For a second parameter, the distribution of the global PIC matched the MAF spectrum globally or across genepools (S3 Fig), since the former is a function of the genotypic classes. Similarly, for a third parameter, the genome-wide nucleotide diversity (π) was bimodal and inflated when computed across genepools, but was unimodal and reduced when computed within genepool (S4 Fig) given the clear genepool divisions for the SNPs [17].

**Fig 4:**
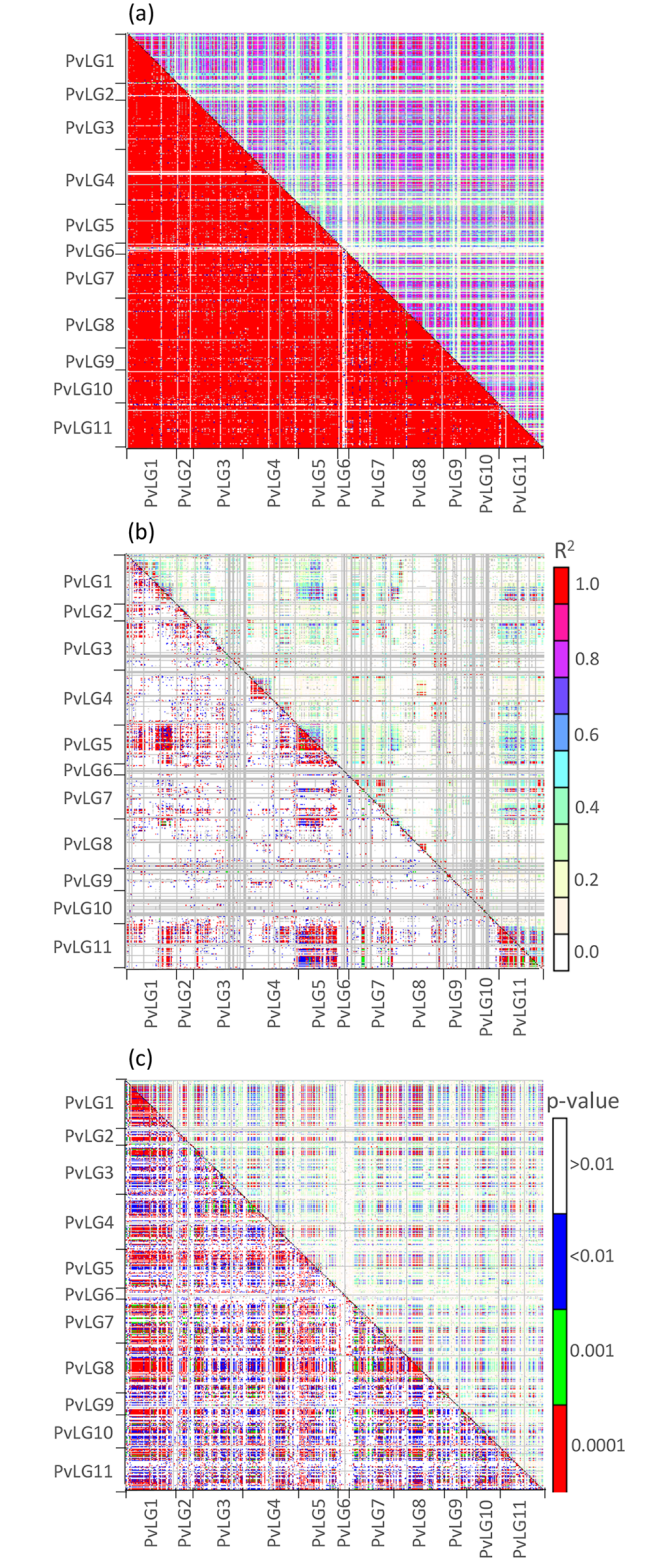
Genome-wide linkage disequilibrium as measured by r^2^ (upper triangles) and its p-value (lower triangles) (a) across genepools, (b) within the Andean genepool, and (c) within the Mesoamerican genepool. Linkage groups are shown in the margins.

**Fig 5:**
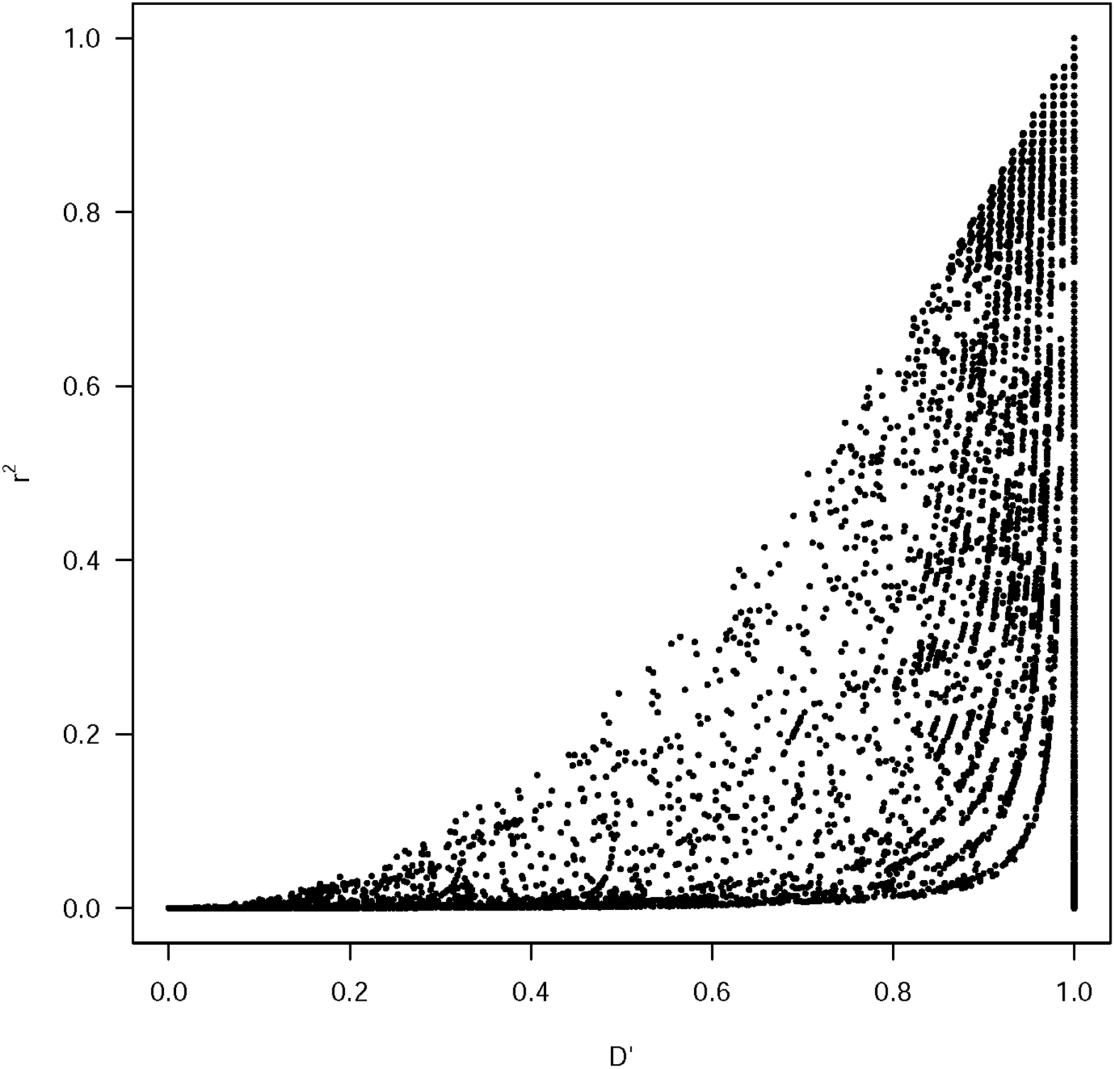
Intra-chromosal decay of linkage disequilibrium (r^2^) as a funcion of genetic distance (cM) (a) across genepools, (b) within the Andean genepool, and (c) within the Mesoamerican genepool. Exponential tendency lines are shown when significant.

LD was also measured on each genepool versus global LD (Fig 4) or randomized LD and found to be more localized on certain linkage groups and reduced within genepools. LD decayed to 0.1 r^2^ within 20cM and 30cM in the Andean and Mesoamerican genepools, respectively (Fig 5). It decayed more rapidly within the Andean genepool likely due to its reduced race substructure. Global intra-chromosomal LD decayed little as a function of genetic (S5 Fig) and physical (S6 Fig) distances.

**Fig 6:**
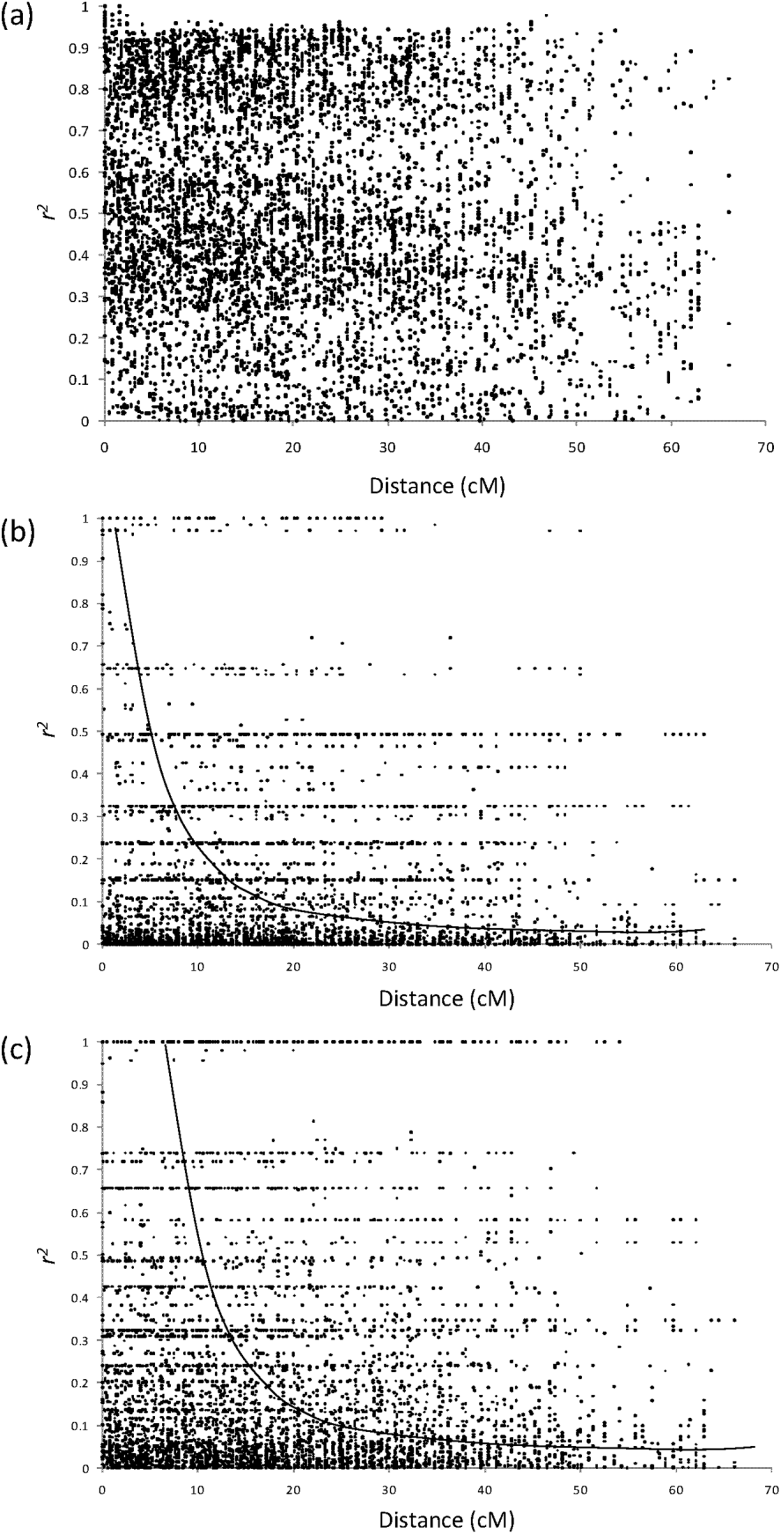
Relationship between D′and r^2^ for the SNP markers.

**Fig 7:**
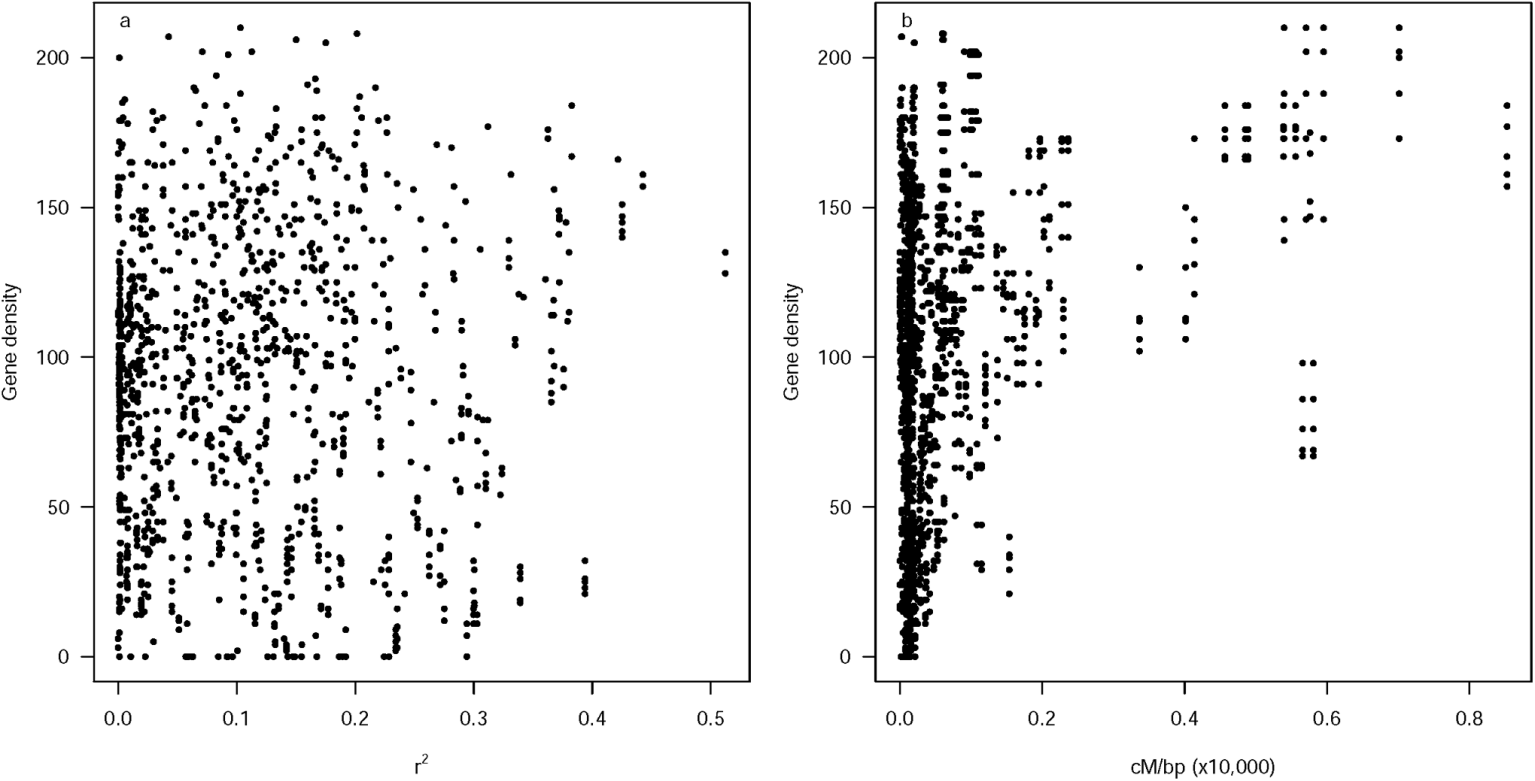
Relationship between gene density and linkage disequilibrium and recombination rate, respectively, measured as r^2^ (a) and genetic to physical distance ratio in cM/bp x 1,000 (b). Both analyses based on sliding window analysis comparing number of genes in a 1 Mb window at 200 Kb interval walk speed throughout the genome.

Overall LD measured as r^2^ and D′ were correlated in all chromosomes (Fig 6) and at each chromosome (S7 Fig). As D′ increased, r^2^ took on any value between 0 and (D′)^2^. Distinction of linkage groups by linkage disequilibrium blocks was only achievable in the analysis carried out within the Andean genepool. On the other hand, inter-chromosomal linkage disequilibrium was more prevalent within the Mesoamerican genepool likely due to its extensive race substructure. When genepool structure was not accounted for, genome-wide linkage disequilibrium was notoriously widespread and did not decay with genetic distance. To study the relationship of R and LD, respectively, with gene density (Fig 7), we conducted a sliding window analysis comparing number of genes in a 1 Mb window at 200 Kb interval walk speed throughout the genome to the r^2^ (%) and recombination rate (cM/bp) value in that window. The relationship was significant in both cases with P = 0.031 and P<0.0001, respectively. Therefore, we can conclude that where the gene density was higher, the recombination rate and r^2^ were higher. This indicated that LD decay was higher in windows that were gene-rich compared to those windows that were gene-poor.

## Discussion

### Features of the new reference map

The TOG based SNPs were useful for creating a saturated genetic map for the BAT93 x Jalo EEP558 population, which has been widely used in previous studies as a reference population [1]. The SNP markers were all gene based in this study which allowed them to be stable and polymorphic in a large number of bean crosses [17] and particularly useful in the core population of BAT93 x Jalo EEP558. Additional SNPs made for Illumina arrays were discovered previously [15] but prior SNP arrays were based more on non-gene based sequences than on gene sequences. This distinction may be important in the conversion of SNPs to other high throughput technologies such as Kaspar or SEquenom assays, where multiple SNPs in a short physical distances within 1 Kb can interrupt the utility of the techniques [16,42].

In both the work of Blair *et al*. [17] and Hyten *et al*. [15], the discovery of inter-genepool SNPs was made with the Andean genotype Jalo EEP558 and the Mesoamerican genotype BAT93. Additional SNP loci have been discovered recently by the method of genotyping by sequencing (GBS) developed as a quick method for genome analysis [43], but their conversion to actual marker assays is still pending for other common beans [22,44,45]. GBS data for individual genomes and even individual mapping populations can vary based on the enzyme used for the reduction in genome representation and the resultant sequence coverage used for SNP detection. Recent studies in common bean genome re-sequencing, have shown that many GBS polymorphisms are actually between paralogous genes rather than alleles of a single gene (Q. Song, pers. Communication) making their validation all the more important.

In our study, observations from the refined genetic map showed the TOG markers were well distributed across and within all the linkage groups with even genetic and physical distances between most markers with the exception of those at chromosome ends. Another observation was that the genetic map was smaller overall than previous maps. This could be explained by the fact that some of the SNP markers were grouped in blocks, making for a more condensed genetic map. Meanwhile, the full genome sequence of *Phaseolus vulgaris* was useful for linkage group to chromosome identification and therefore marker orientation. For example the isozyme or phenotypic markers for BCMV resistance (*I* gene) and enzyme *Chs* mapped to the correct locations on Pv2 as did the loci for isozymes *Aco*2 and *Diap* on Pv5 and the locus for *Chi* and the seed protein phaseolin (*Phs*) on Pv7 according to original mapping [29], for this same population of RILs. The SSR marker locations from Blair *et al*. [30] for the BAT93 x JaloEEP 558 population and for comparative mapping with DOR364 x G19833 RILs were confirmed by the physical map. The Cook lab map for the TOG markers was placed into the LIS database before availability of the Schmutz et al. [5] genome sequences and therefore did not have chromosome identification associated with each linkage group but the full Blair lab map has been deposited in LIS as well with correct chromosome identification and physical links.

In the final genetic mapping the number of SNP markers varied between linkage groups. The number of total markers per linkage group ranged up to 145 for Pv2 (49.0 Mb) with more than 110 markers on Pv1 (52.2 Mb in length) and Pv3 (52.3 Mb). Meanwhile, linkage groups Pv4 (45.8 Mb), Pv5 (40.7 Mb) and Pv10 (42.2 Mb) were low in SNP marker saturation with 22, 40 and 27 TOGs, respectively. The remaining linkage groups had similar numbers of markers ranging from 61 on Pv11 (50.2 Mb) to 88 on Pv7 (51.7 Mb). A chi-square test (significance P≤0.05) showed that the distribution was not equal between linkage groups for the SNP markers. In contrast the RFLP and SSR markers were more evenly distributed as anchors across all linkage groups (*Χ*^2^ = P≥0.05). We analyzed the SNP distribution further in two ways: 1) by comparing linkage groups and 2) by comparing regions within the physical map for each chromosome.

In the first of these analyses, the density and distribution of SNP plus legacy markers compared well to the analysis of another central mapping population for common bean as described previously [22]. These authors evaluated an inter-genepool RIL population to map 513 GBS based SNP loci over 943 cM genetic distance covering 95% of the common bean physical map with 26 to 65 unique markers per chromosome. Interestingly the linkage group sizes in our study and theirs were correlated (r = 0.78, P>0.001) with Pv4, Pv5, Pv6, Pv9 Pv10 and Pv11 being smaller than the other linkage groups especially Pv2, Pv3, Pv07 and Pv8. In one difference, Pv1 was larger in our genetic map then in that of Bhakta *et al*. [22]. The only previous use of the BAT93 x JaloEEP 558 population for SNP based markers was by McConnell *et al*. [46] but with fewer markers used to determine synteny relationships between legumes.

The origin of the TOG SNP markers used in our study is similar to the *Pst*I methylation sensitive source of markers in Bhakta *et al*. [22]. In both cases high GC content, gene-rich regions of the chromosome ends (euchromatin) were more highly represented on the genetic map than high AT content, non-gene regions around the centromeres (heterochromatin), and this was borne out by genetic and physical mapping in our study and that of Bhakta *et al*. [22]. These authors, like us, also identified regions of pCENR that had highly suppressed recombination and the alignment of the centromeric regions on our two maps is very similar providing evidence for the accuracy of their GBS methodology based on our more time-tested Illumina bead methodology. Common bean is predicted to have mostly acrocentric chromosomes [47], and this is supported by our findings.

Genetic to physical map comparisons showed that almost all the linkage group / chromosome plots could be fit by sigmoidal curves except in two cases: Pv09 (37 Mb in length) was telocentric, as predicted by Bhakta *et al*. [22] due to the presence of large blocks of 45S and 5S ribosomal DNA. This agreed with our finding of no plateau in the Mb/cM comparison for this chromosome. In addition, the very short Pv06 (31.9 Mb) had an L-shaped curve showing a region of low recombination from the physical distances of 5 Mb to 15 Mb followed by high recombination rates from 15 Mb to the chromosome end. The average recombination rate for Pv6 was 1.86 Mb/cM, but could be divided into two segments: a plateau with recombination rate of 5 Mb / cM and another region of 0.25 Mb / cM. The whole genome had an average physical to genetic map ratio of 2.13 cM / Mb, but each scatter plot showed a plateau region with almost no recombination (pCENR regions) and adjoining slope regions on the sigmoidal curves (equivalent to chromosomal arms). The ratio per chromosome was not associated with physical size and was fairly constant ranging with an average of 2.04 ± 0.53 cM / Mb.

Our genetic mapping results compared favorably with those of the common bean genome sequencing and re-sequencing study of Schmutz *et al*. [5] or the non-gene SNP markers of Song *et al*. [20]. In those studies a genetic map was created for an F2 population from a Stampede (Mesoamerican) × Red Hawk (Andean) cross. The genetic map of Song *et al*. [20] was correlated with our linkage group sizes (r = 0.58) and with the physical length from Schmutz et al. [5] but to a lesser degree (r = 0.56) than with Bhakta et al. [22], perhaps because the latter authors used a RIL population like us, rather than an F2 mapping population. Similarly the correlation between chromosome size and number of SNPs per linkage group in Hyten *et al*. [15] was moderate (r = 0.43). The relevance of these correlations is in helping to determine which SNP markers are most appropriate to use for full genome coverage.

In summary regarding the linkage analysis, we presented a saturated genetic map with well characterized SNPs on a reference population for common bean, derived from the cross BAT93 x Jalo EEP558, and we used this map for estimating recombination rates and LD decay across the genome. This genetic map has been useful in determining recombination rates among many types of markers. As a core mapping population, the BAT93 x JaloEEP 558 set had many comparable markers with other highly saturated genetic maps such as those of Córdoba *et al*. [48] and Galeano *et al*. [42]. As conclusions from the inter-genepool maps studied so far, pericentromeric regions had highly repressed recombination while TOG markers were found in areas of high recombination regions. Our genetic map had the difference with the Stampede x Red Hawk map of being gene-based and derived from recombinant inbred lines, which allowed us to concentrate on estimating recombination rates after multiple generations of inbreeding. It will be very interesting to have comparisons of regional rates of recombination in genetic maps of different types of recombinant inbred line populations especially comparing those from intra-genepool versus inter-genepool crosses. In the meantime the genetics map presents useful SNP markers for common bean breeding such as those around the *I* gene for virus resistance on Pv02.

### Estimates of linkage disequilibrium across the common bean genome

LD estimates were made for the same SNP markers that were genetically mapped above and for both Andean and Mesoamerican genotypes from Blair *et al*. [17], which are a publically available germplasm preserved by the FAO treaty on genetic resources. Once we considered the effect of genepool on LD, the rates of LD decay were fairly typical of a self-pollinating species [10,11]. The results on the diversity panel showed clear genepool differences with much of LD explained by population structure.

A further result of our study was that LD was stronger and decayed slower within the Mesoamerican genepool, likely due to its more extensive race substructure. This is interesting because a bottleneck for the Andean beans, as has been speculated by arguing a Mesoamerican origin of the common bean [7], would imply higher LD within the Andean genepool [2,49]. A similar difference was found when comparing genepool specific versus global marker associations in a panel of Brazilian genotypes [50]. In that study, 80% of loci comparisons had significant LD when the entire group of cultivated beans were considered but only 8 and 23%, respectively when the Andean or Mesoamerican genepools were considered separately. It was notable that like two other early studies of LD in common bean by Blair *et al*. [4] and by Kwak and Gepts [51]. The Brazilian study [50] found similar overall patterns of population structure effect on LD values. Andean levels of LD were lower than for Mesoamericans based on the distinctiveness of the Durango-Jalisco versus Mesoamerica divide being greater than Nueva Granada / Peru race differences, thus validating results with sequenced gene analysis by Bitocchi *et al*. [7]. The ability to detect LD is affected by genepool [39], sub-species and species divergence [52]. Domestication bottlenecks and selective sweeps around certain adaptive or selected genes affect overall and locus specific LD rates, especially in legumes [10].

A final point for our study was that the rate of LD decay was low when marker associations were studied within linkage groups and for each genepool separately. More focused studies of LD at certain loci have shown that LD decay was moderate and variable around drought tolerance genes [24–26] across the APA family of insect resistance genes and pseudogenes [23]. The study of LD in euchromatin rather than in heterochromatin, estimated to make up ~54% of the genome [5], is useful for plant breeders of this inbreeding crop.

Within Andean LD analysis is likely to show higher recombination than within Mesoamerican genepool mapping due to less population structure and potentially higher polymorphism. LD rates have been shown to affect the potential for QTL and association mapping of traits in common bean. Given the higher within-Andean LD, initial successes with genome wide association have been for Andean studies [27,53,54]. More challenges for association will be likely in the Mesoamerican genepool but recent evidence with a North American panel shows promise [19,55] while other recent studies by Zuiderveen et al. [56] and Perseguini et al. [57] have evaluated association of markers for disease resistance in Andean and Brazilian germplasm, respectively. Since all these studies have focused on harder-to-obtain breeding lines, rather than purely gene bank accessions like we have, they are more country and site specific than our work. Our emphasis on germplasm entries that are in the CGIAR international system for genetic resources makes our study a baseline study for future association analysis in multiple countries since the germplasm can be easily obtained through the FAO International Plant Genetic Resources treaty rather than individual laboratories.

## Supporting information

S1 Table Genotypes in the linkage disequilibrium (LD) germplasm panel including 71 Andean and 115 Mesoamerican genotypes from Blair *et al*. (2013). (DOCX)

S2 Table TOG marker names, map positions, and flanking sequences. (XLSX)

S3 Table Homology of legume TOG markers mapped in this study. (XLSX)

S1 Fig Genetic map based on BAT93 x JaloEEP558 recombinant inbred line population for all 11 common bean (Pv) chromosomes showing distances (cM) to the left and markers to the right, respectively. (PDF)

S2 Fig SNP marker location comparisons on linkage map distance (cM, y-axis) and physical distance (Mb, x-axis) across each of the eleven chromosomes of common bean (Pv) showing a dashed line at the position of the centromeres according to Schmutz *et al*. (2014). (PDF)

S3 Fig Distribution of minor-allele-frequency (MAF), also known as folded site frequency spectrum, for 768 single nucleotide polymorphism (SNP) markers used in this study (a) across genepools, (b) within the Andean genepool, and (c) within the Mesoamerican genepool. (PDF)

S4 Fig Genome-wide distribution of nucleotide diversity (π) values for 768 single nucleotide polymorphism (SNP) markers used in this study (a) across genepools, (b) within the Andean genepool, and (c) within the Mesoamerican genepool. (PDF)

S5 Fig Intra-chromosal decay of linake disequilibrium (r^2^) as a function of genetic distance (cM) for pairwise comparisons of the SNP markers on each chromosome (Pv). (PDF)

S6 Fig Intra-chromosal decay of linake disequilibrium (r^2^) as a function of physical distance (Mb) for pairwise comparisons of the SNP markers at each chromosome (Pv). (PDF)

S7 Fig Per chromosomal (Pv) relationship between D′and r^2^ for the SNP markers. (PDF)
