# Supplementary material for: Uneven recombination rate and linkage disequilibrium across a reference SNP map for common bean (Phaseolus vulgaris L.)
Source: PLoS One. 2018 Mar 9;13(3):e0189597. doi: 10.1371/journal.pone.0189597 (PMC5844515; doi:10.1371/journal.pone.0189597)
Supplement: S1 Table — (DOCX) [file pone.0189597.s001.docx]

**Supplemental Table 1.** Genetic mapping and map distance in centiMorgans for each linkage group in the BAT93 x Jalo EEP558 population made up of SNP markers from the trans-legume orthologous genes (TOG) series combined with other types of markers, including restriction fragment length polymorphism (RFLP) markers of the Bng and D series, simple sequence repeat (SSR) markers and isozyme, protein or phenotypic markers.

|  | **First step** | | **Second Step** | | | | | |
| --- | --- | --- | --- | --- | --- | --- | --- | --- |
| **Linkage**  **Group** | **Total**  **Markers in LG** | **Final**  **Distance**  **(cM)** | **TOG SNP**  **markers** | **RFLP-**  **markers** | **SSR-**  **markers** | **Isozyme, Protein**  **Phenotypic** | **Total high LOD**  **markers** | **Final**  **Distance**  **(cM)** |
| Pv1 | 104 | 230.1 | 79 | 7 | 2 | 0 | 88 | 147.0 |
| Pv2 | 145 | 374.5 | 93 | 6 | 3 | 2 (Chs,I) | 104 | 140.6 |
| Pv3 | 119 | 191.1 | 77 | 7 | 0 | 0 | 84 | 114.4 |
| Pv4 | 37 | 105.1 | 22 | 4 | 3 | 1 (Me) | 30 | 84.7 |
| Pv5 | 62 | 103.9 | 40 | 6 | 1 | 2 (Aco2,Diap) | 49 | 82.8 |
| Pv6 | 101 | 171.4 | 78 | 4 | 3 | 0 | 85 | 83.7 |
| Pv7 | 82 | 104.9 | 88 | 6 | 3 | 2 (Chi,Phs) | 99 | 108.3 |
| Pv8 | 93 | 160.9 | 84 | 6 | 1 | 0 | 91 | 99.2 |
| Pv9 | 90 | 72.3 | 77 | 4 | 2 | 0 | 83 | 46.5 |
| Pv10 | 43 | 158.6 | 27 | 3 | 2 | 0 | 32 | 72.2 |
| Pv11 | 89 | 89.7 | 61 | 6 | 0 | 0 | 67 | 67.5 |
| **TOTAL** | **981** | **1762.5** | **726** | **59** | **20** | **7** | **812** | **1097.5** |
