# Supplementary figures and images for: Uneven recombination rate and linkage disequilibrium across a reference SNP map for common bean (Phaseolus vulgaris L.)
Source: PLoS One. 2018 Mar 9;13(3):e0189597. doi: 10.1371/journal.pone.0189597 (PMC5844515; doi:10.1371/journal.pone.0189597)

**b01h**  
147.0 cM

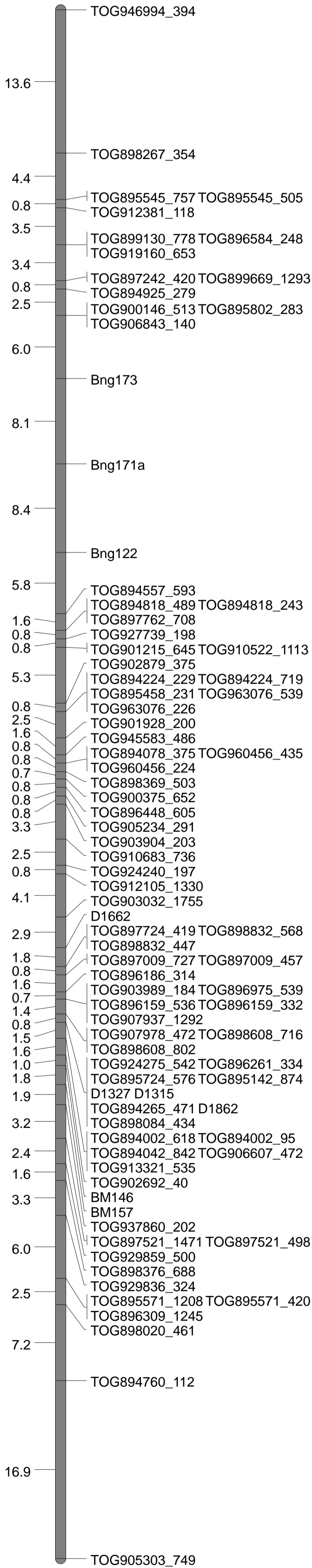

**b02d**  
140.6 cM

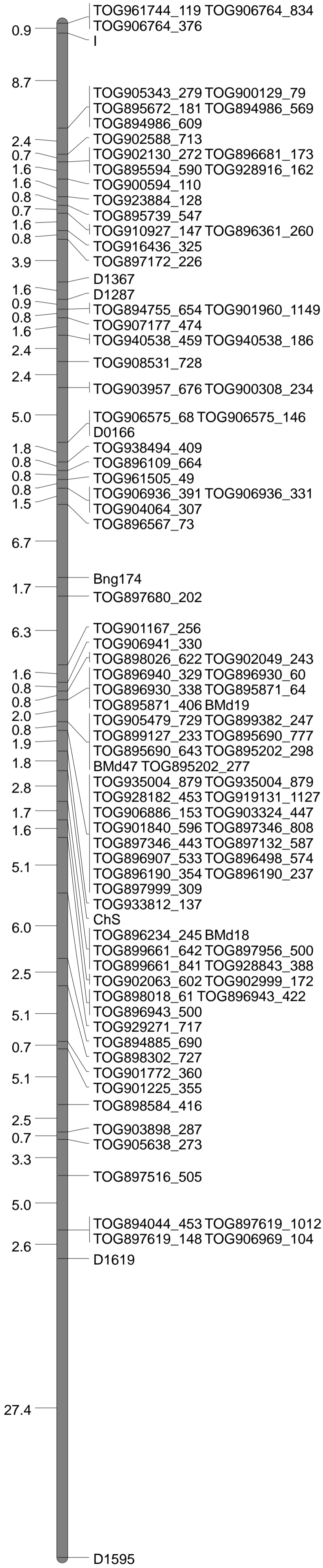

**b03c**  
114.4 cM

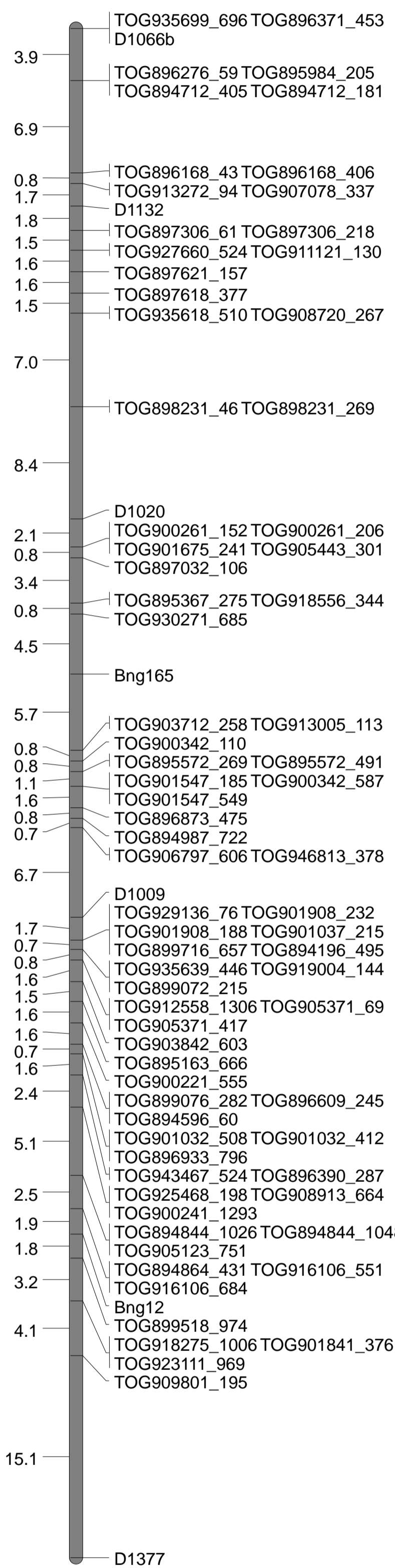

**b04c**  
84.7 cM

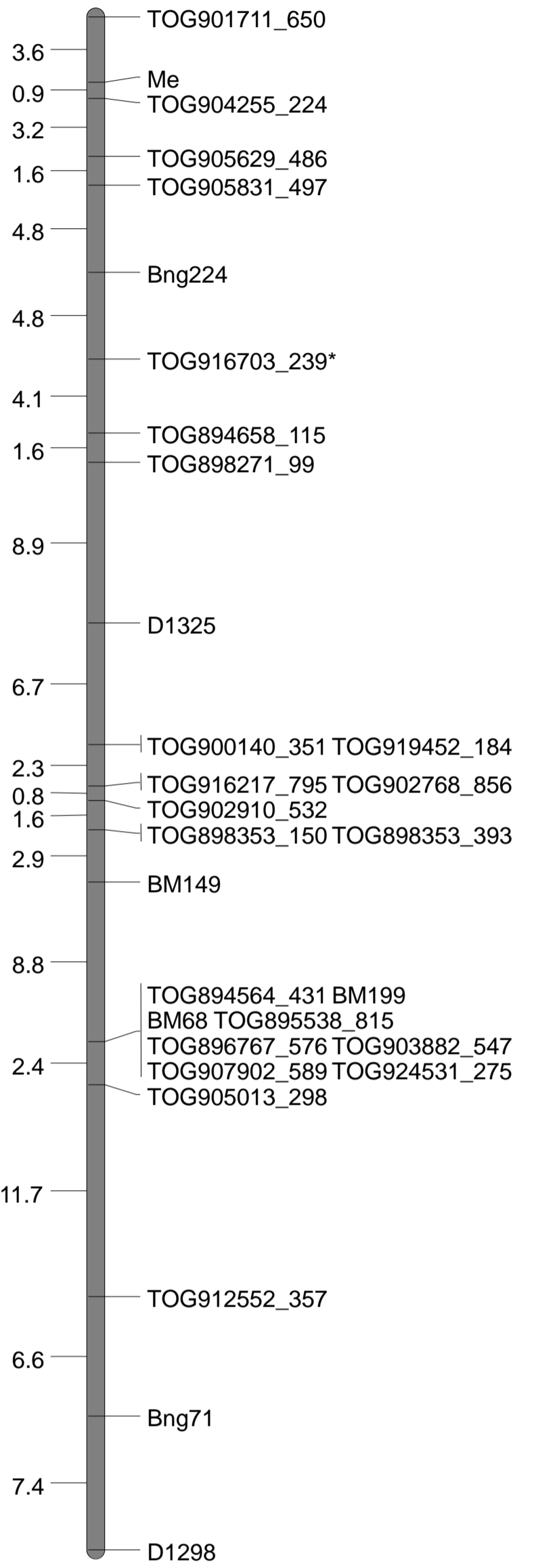

**b05e**  
**82.8 cM**

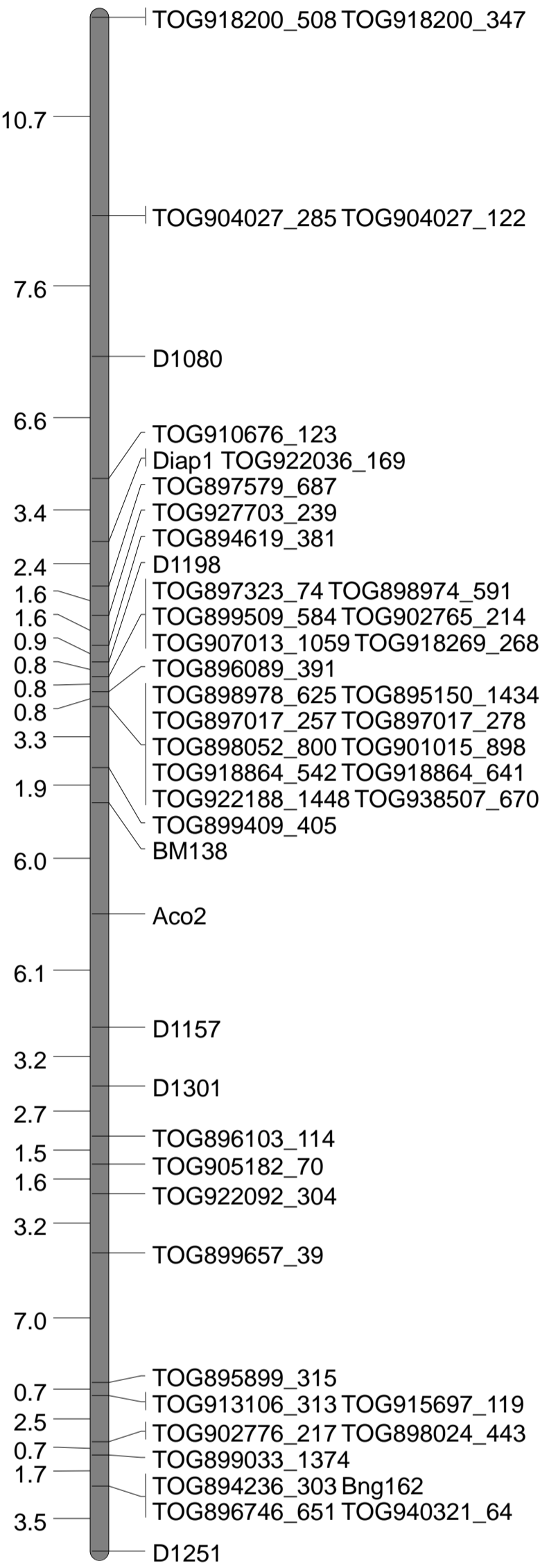

**b06g**  
**83.7 cM**

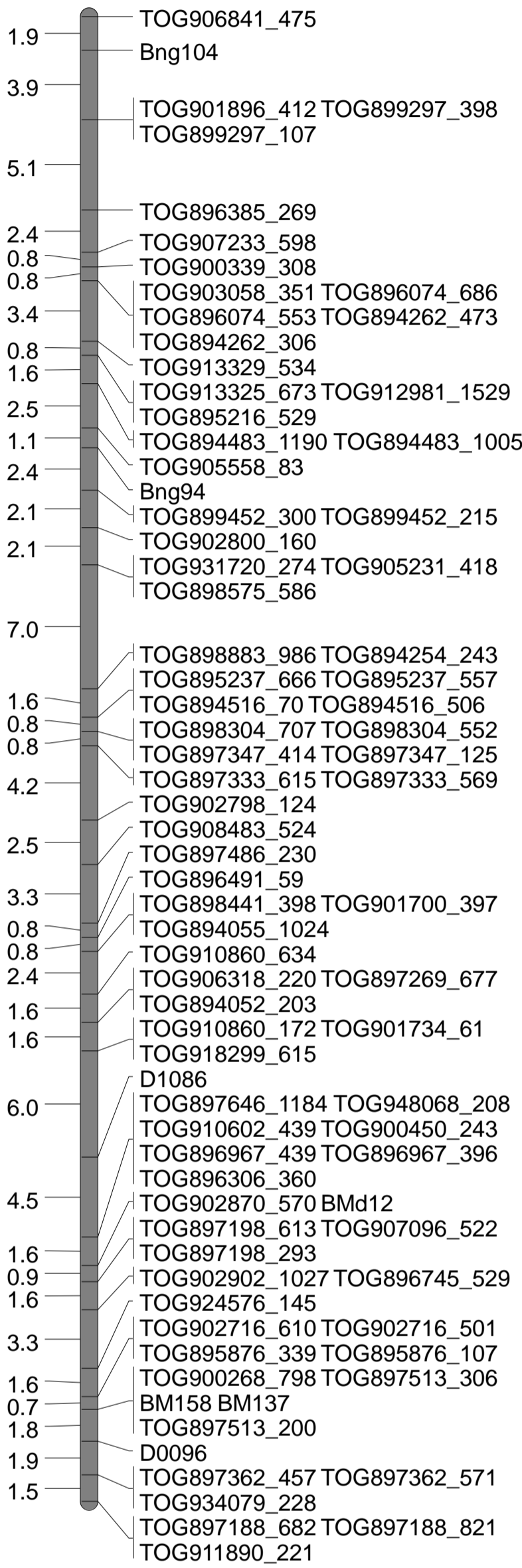

**b07a**  
**108.3 cM**

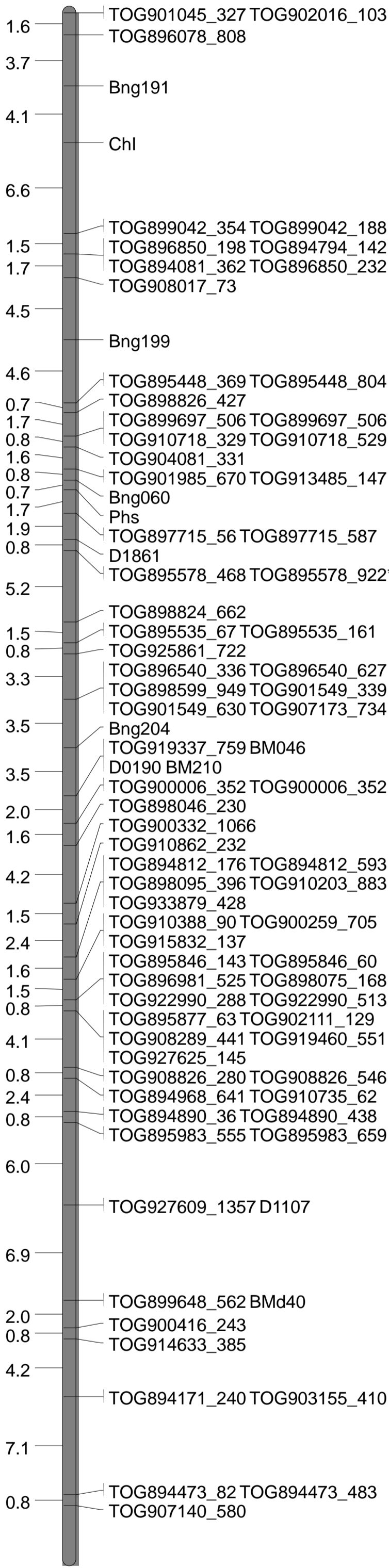

**b08f**  
**99.2 cM**

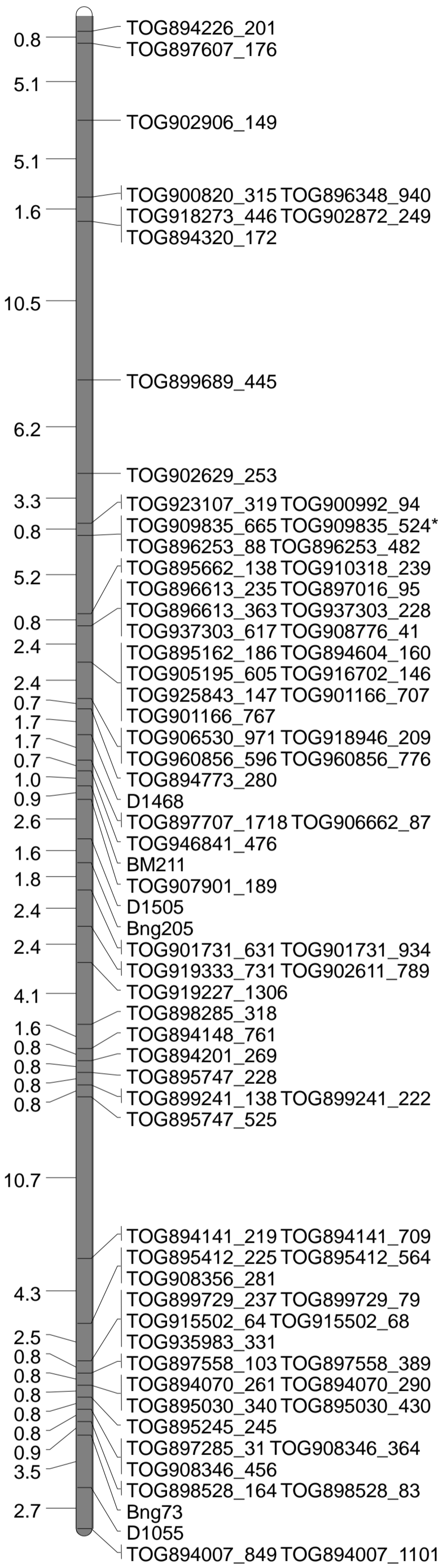

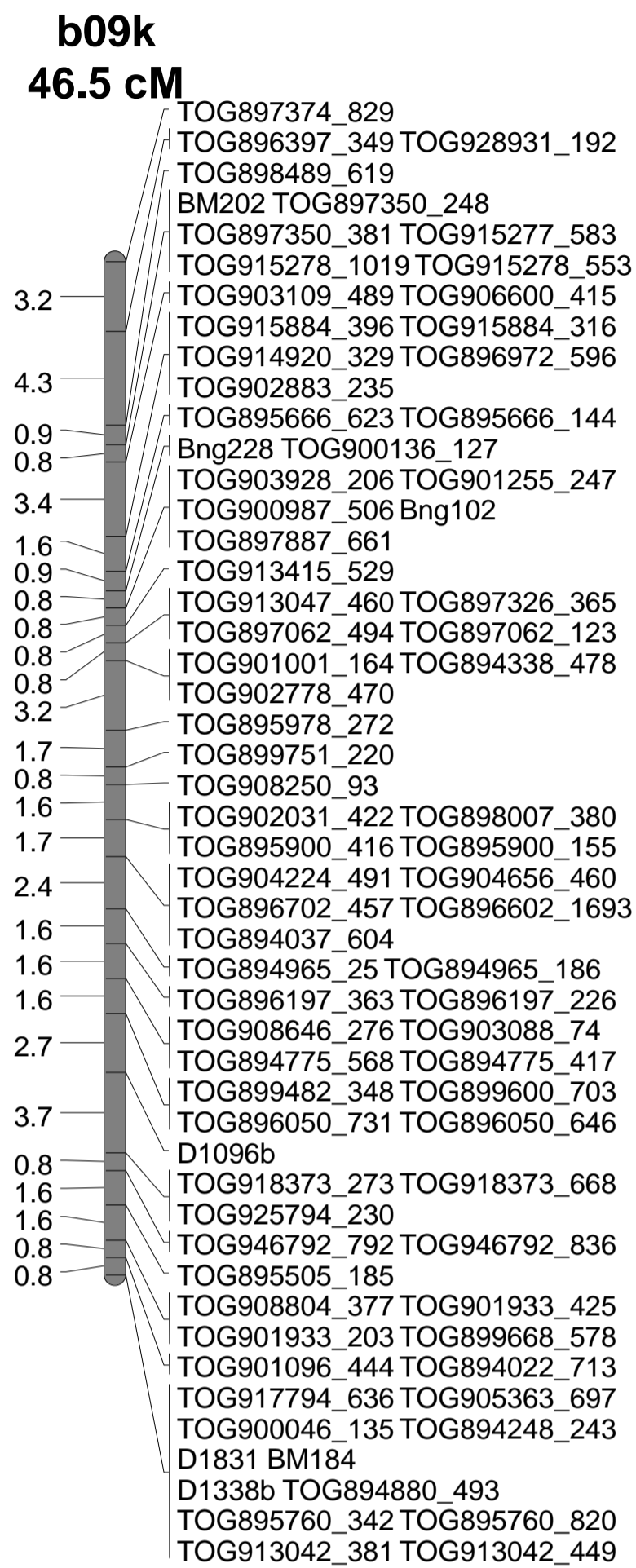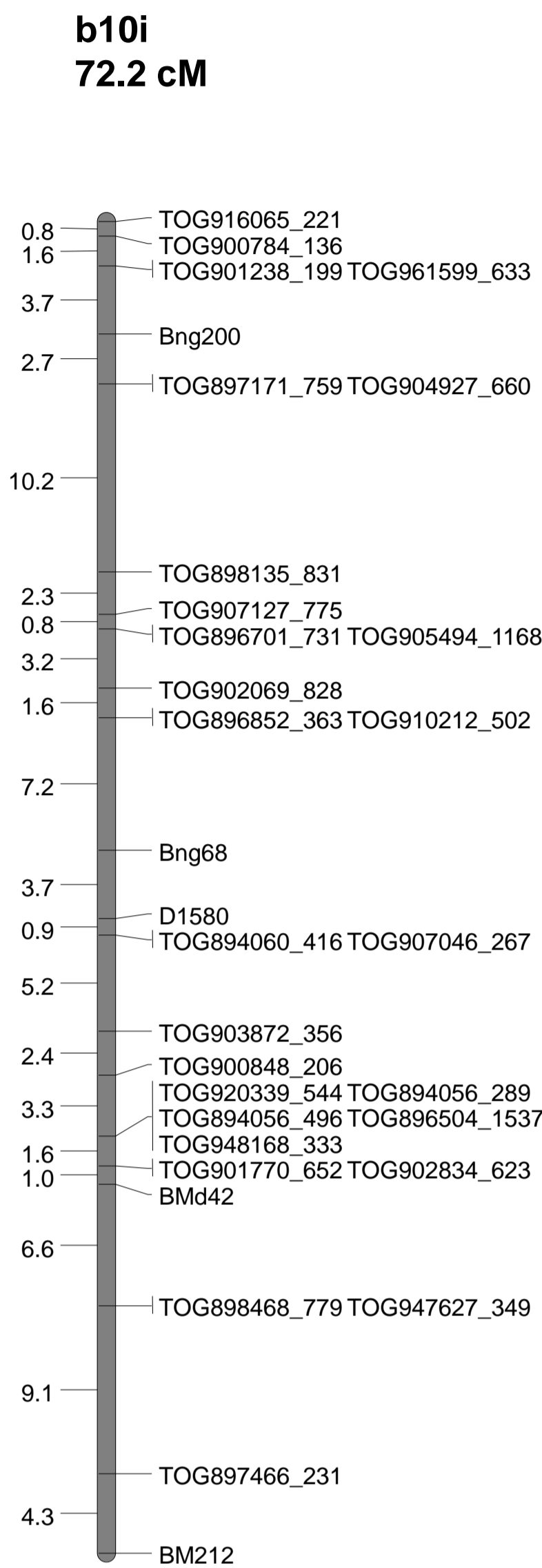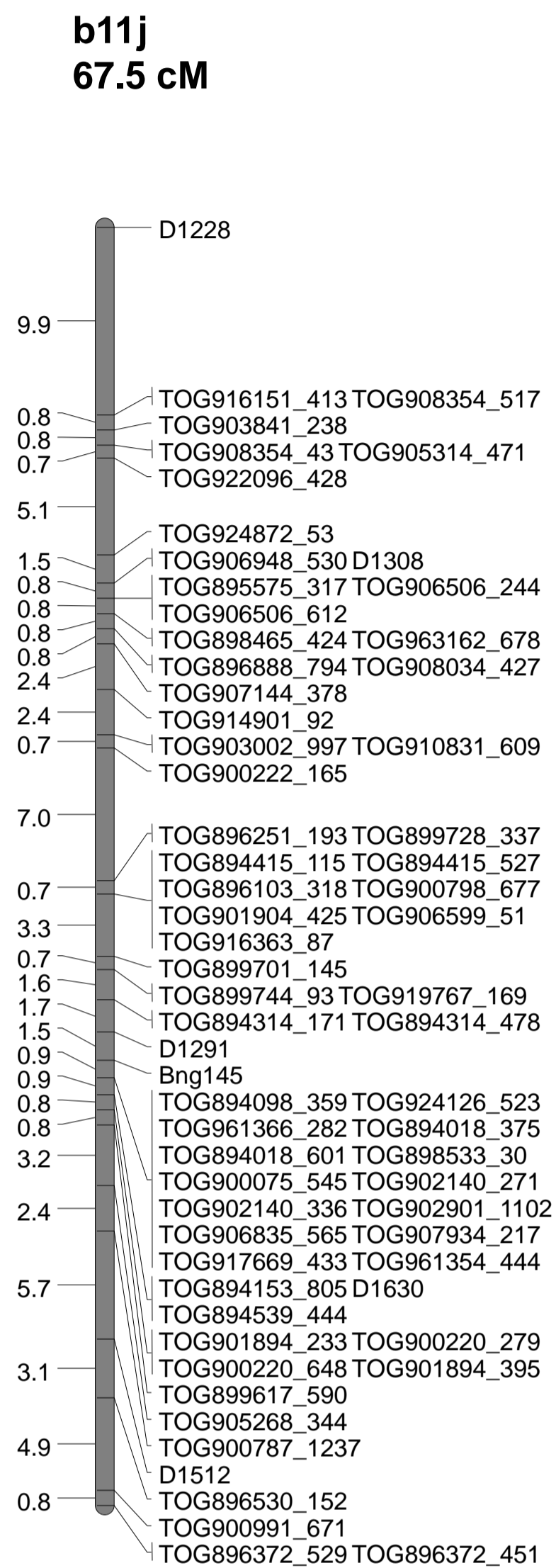

Supplement: S1 Fig — (PDF) [file pone.0189597.s004.pdf]

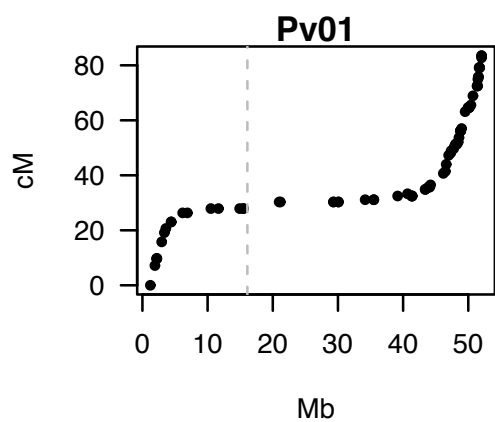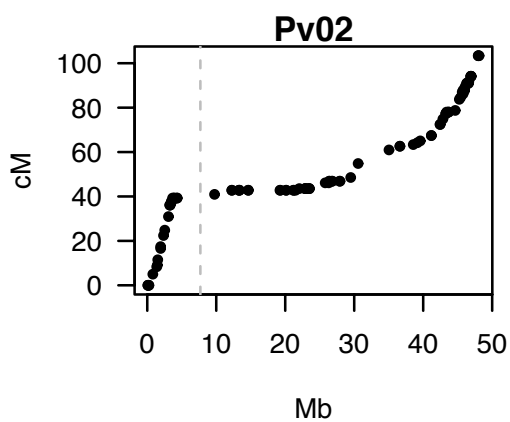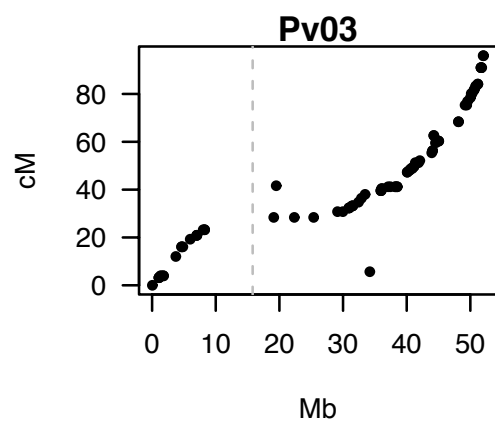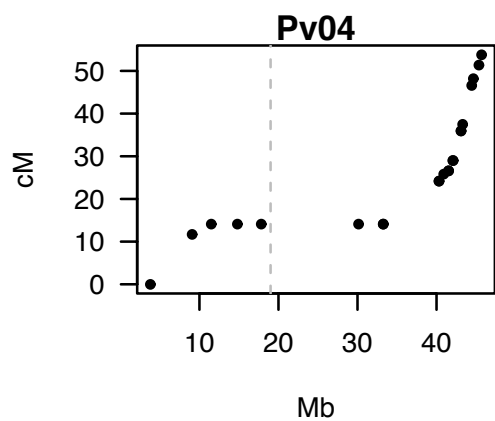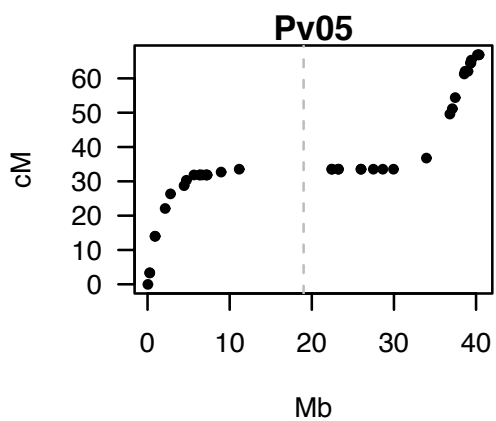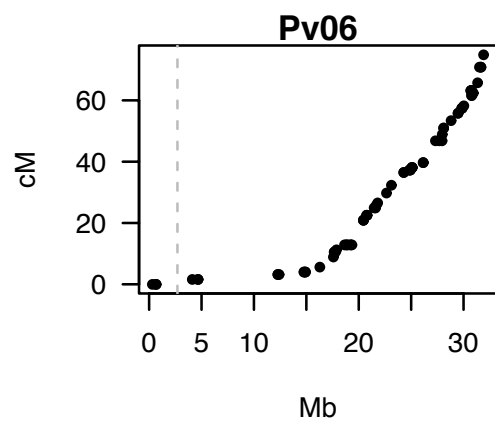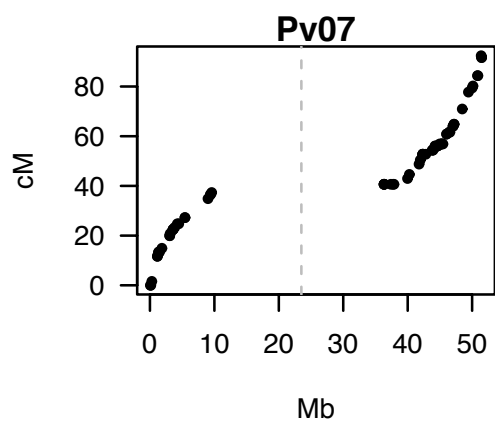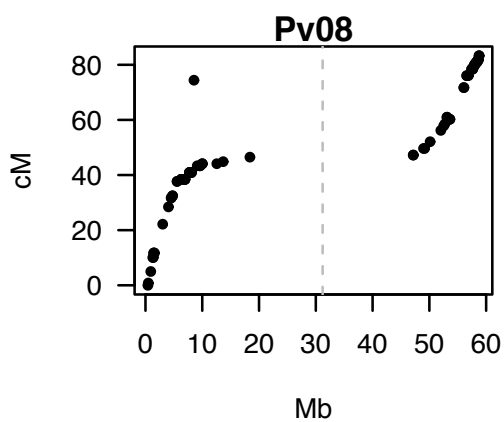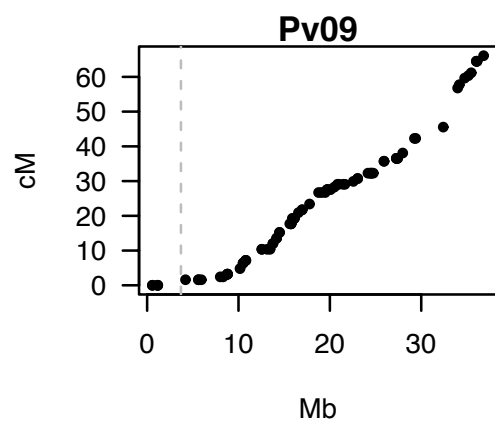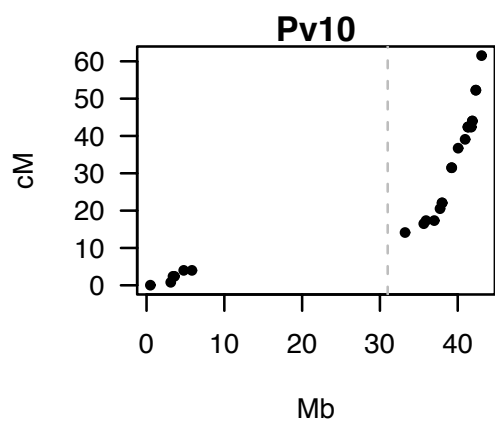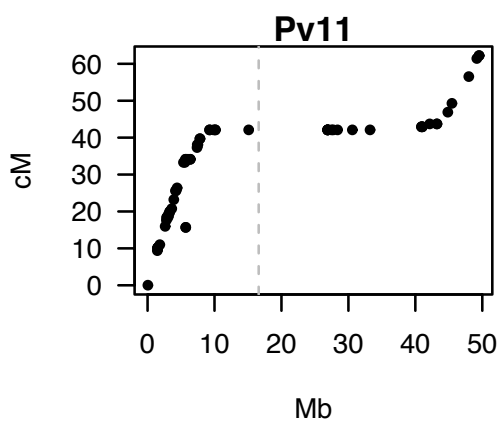

Supplement: S2 Fig — (PDF) [file pone.0189597.s005.pdf]

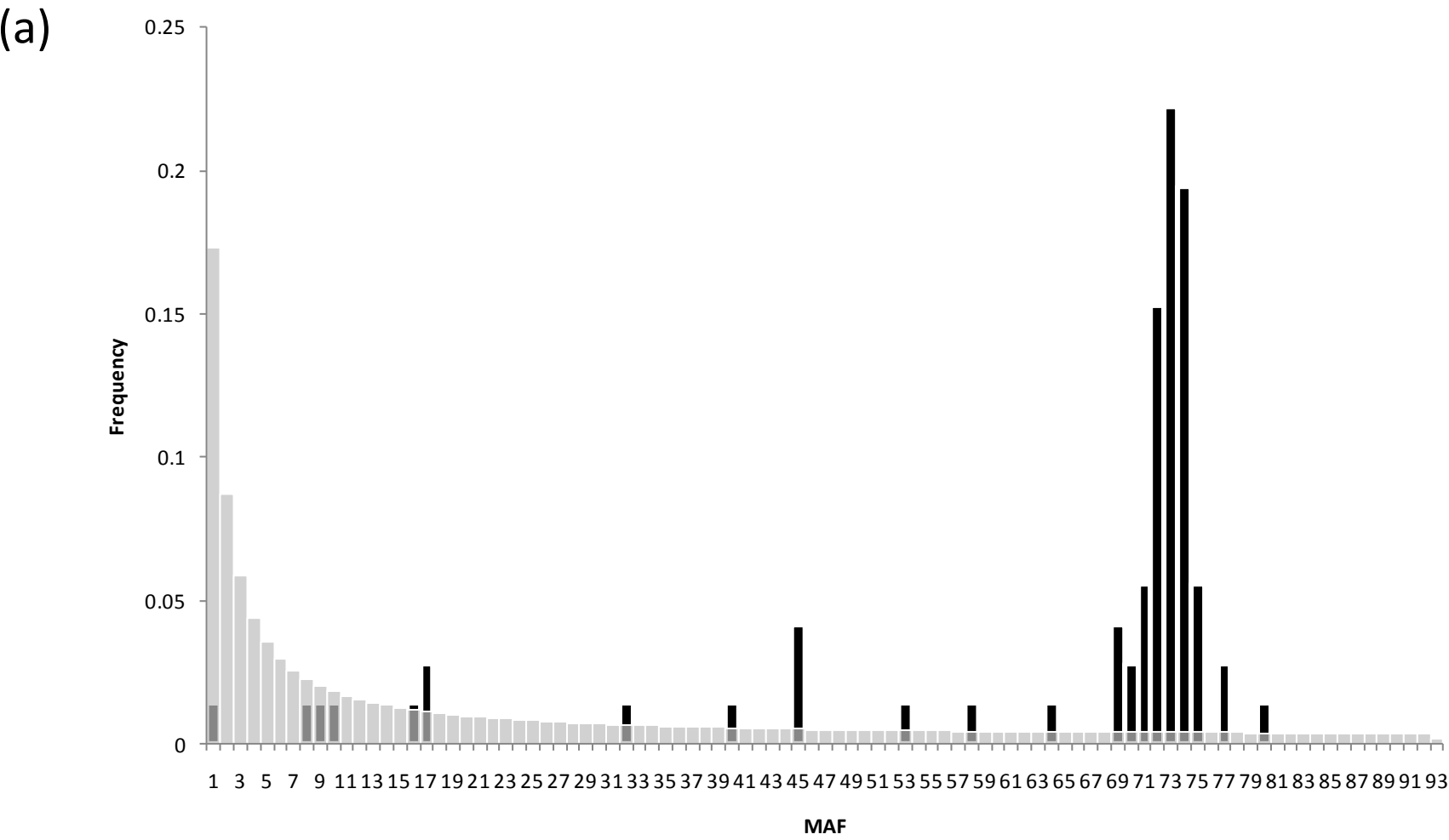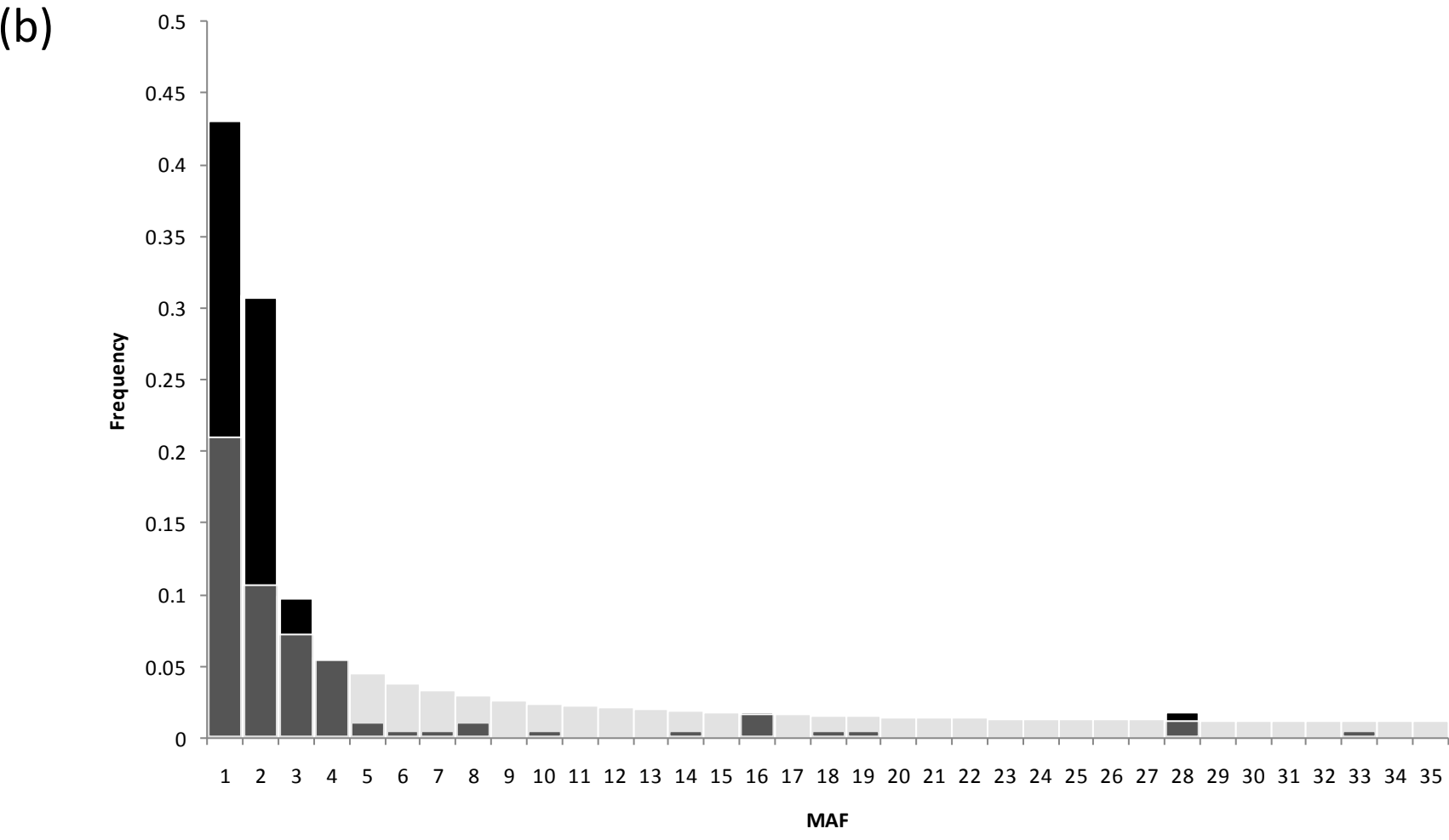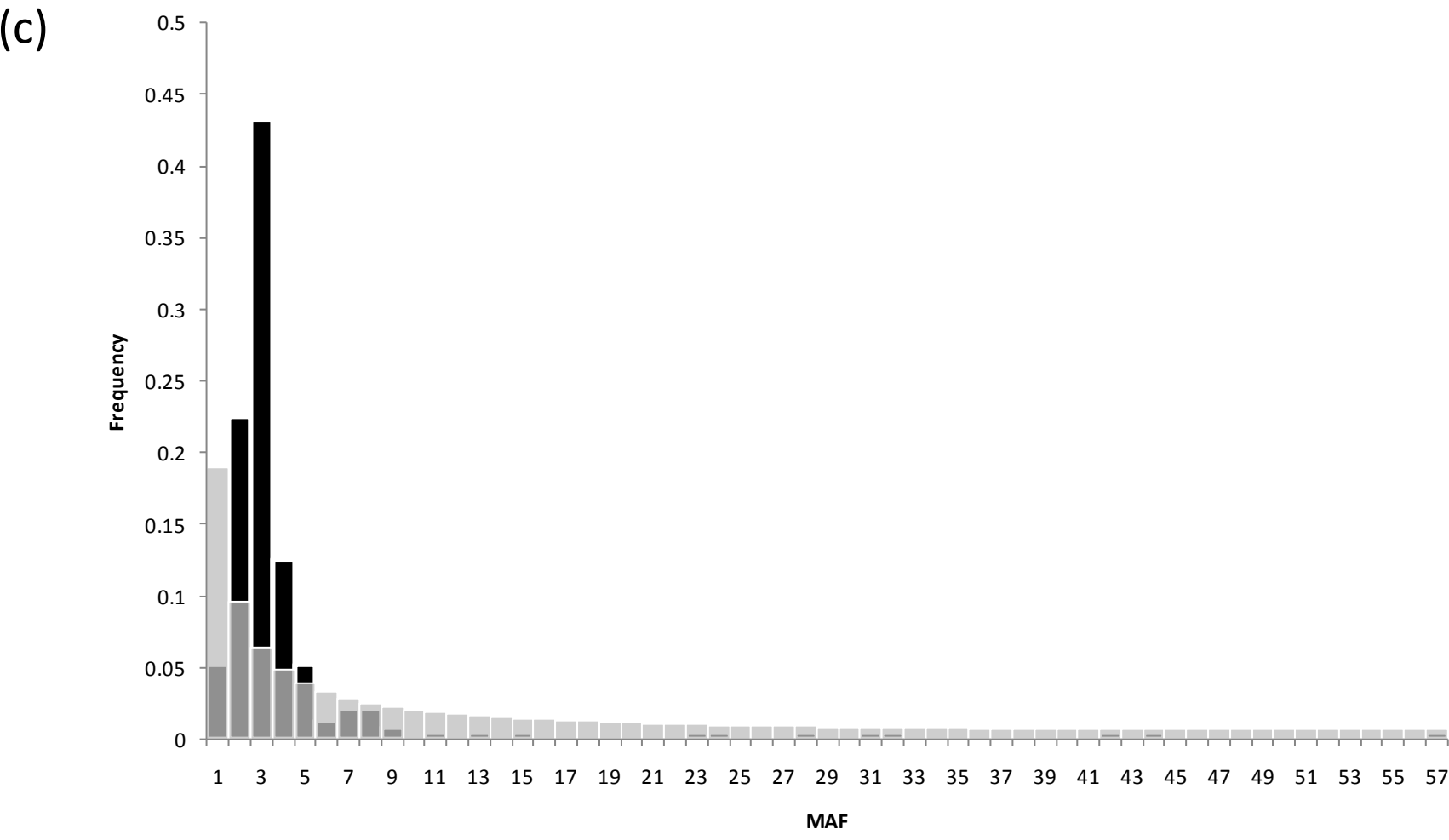

Supplement: S3 Fig — (PDF) [file pone.0189597.s006.pdf]

(a)

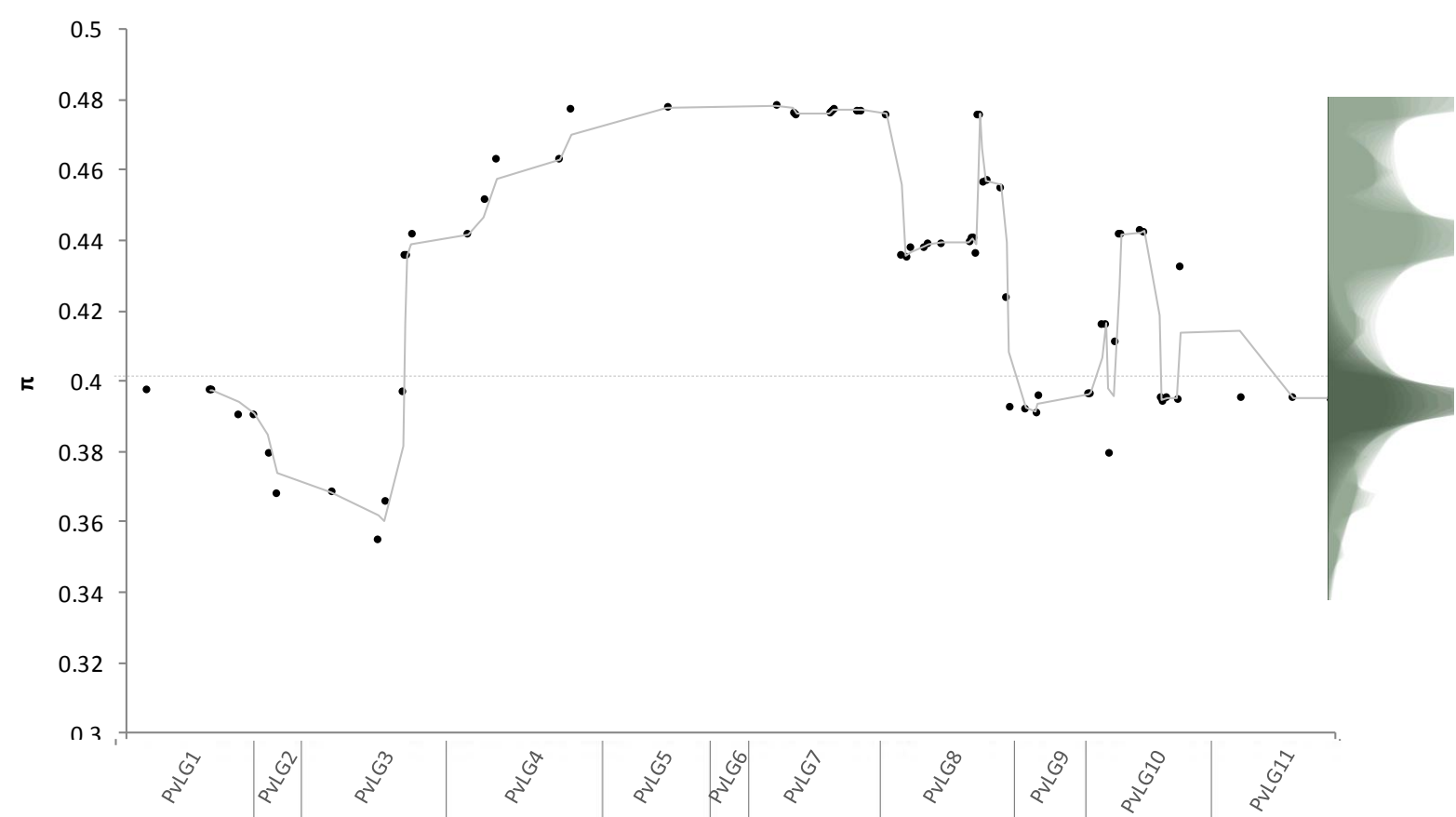

(b)

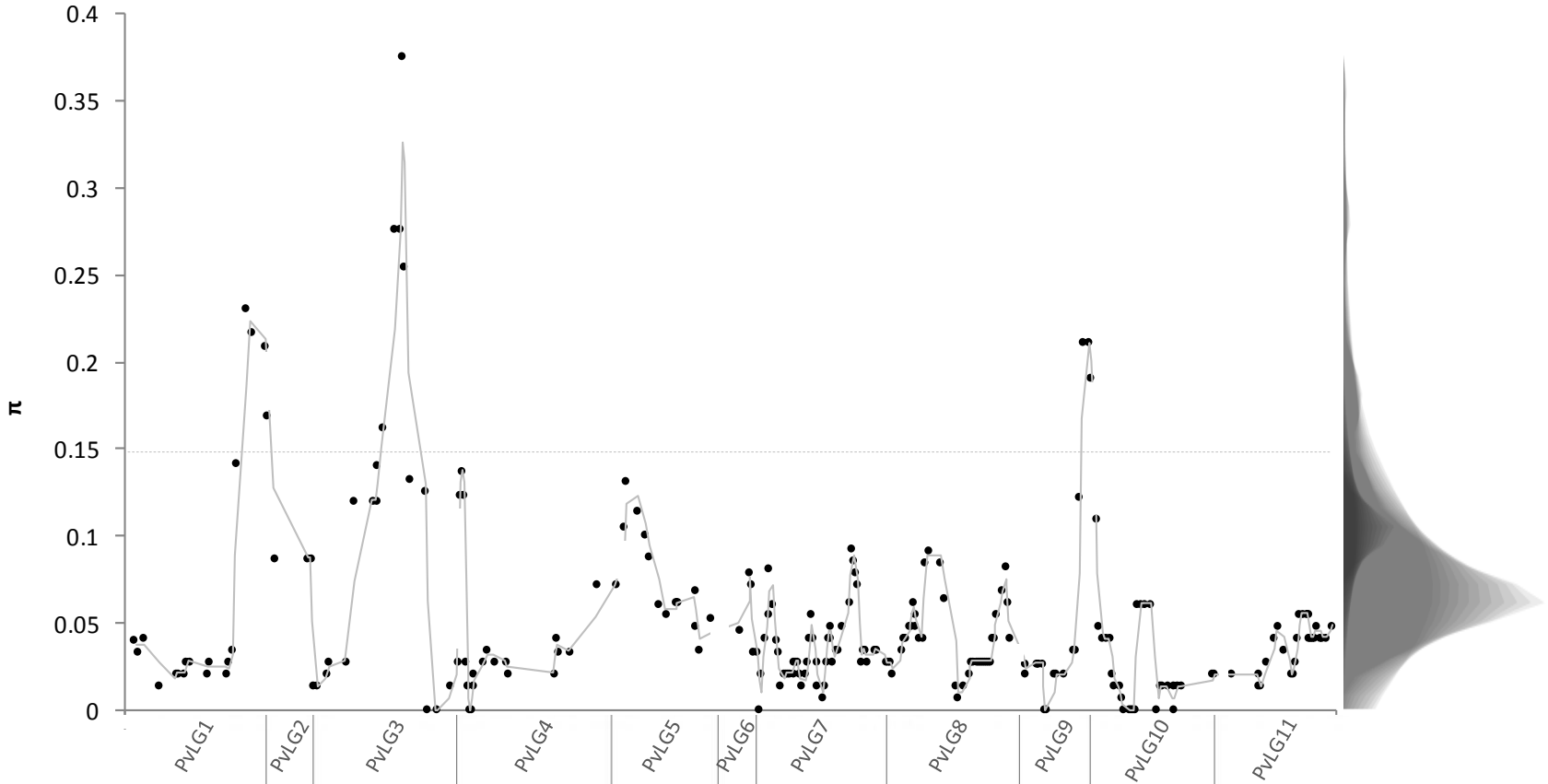

(c)

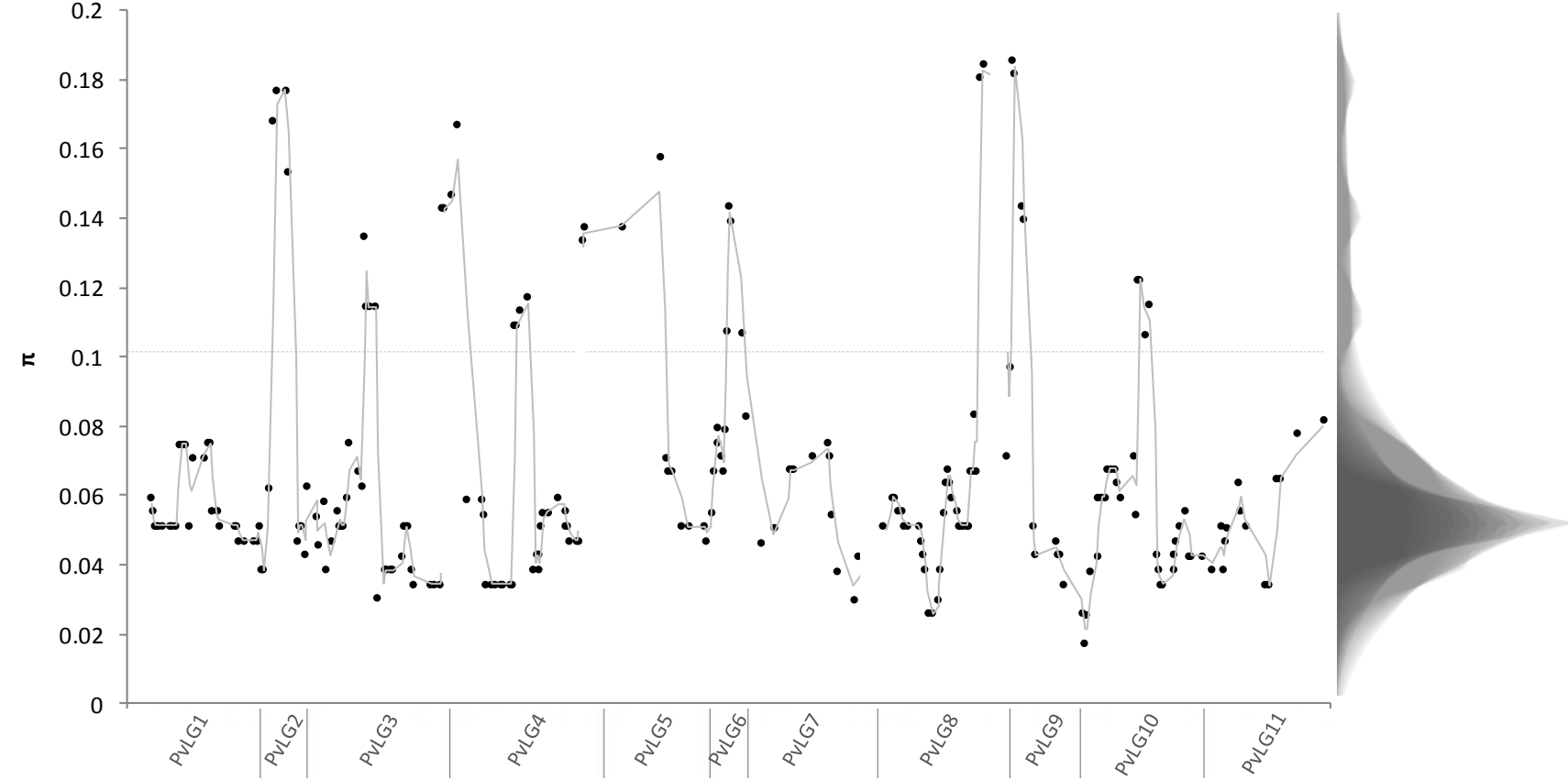

Supplement: S4 Fig — (PDF) [file pone.0189597.s007.pdf]

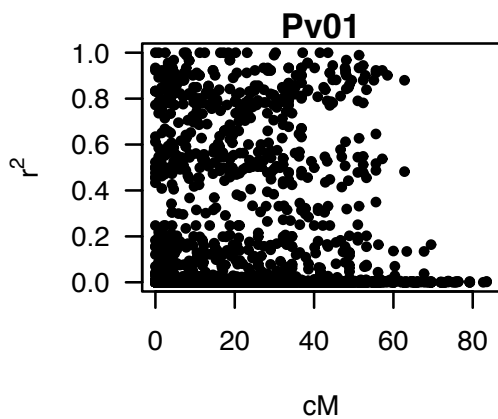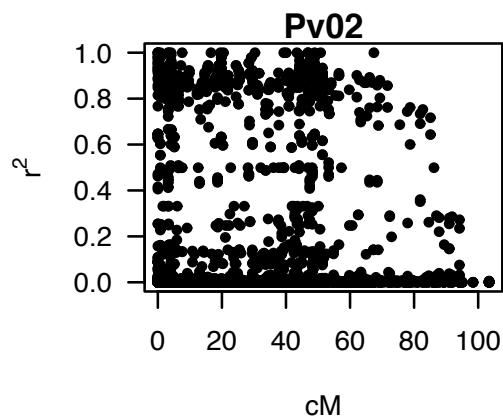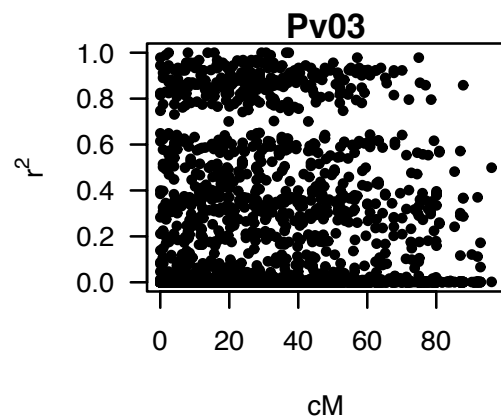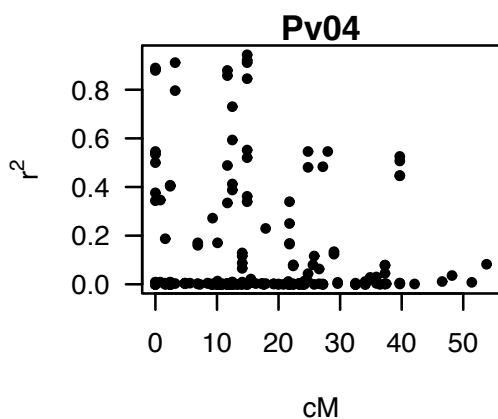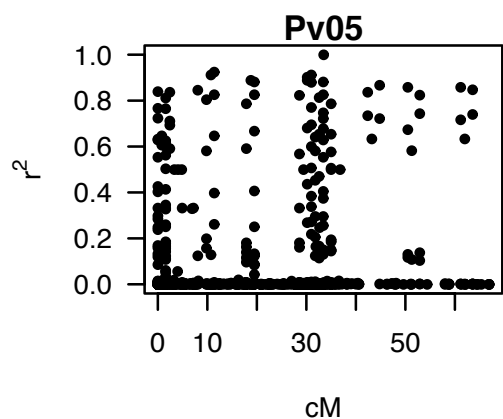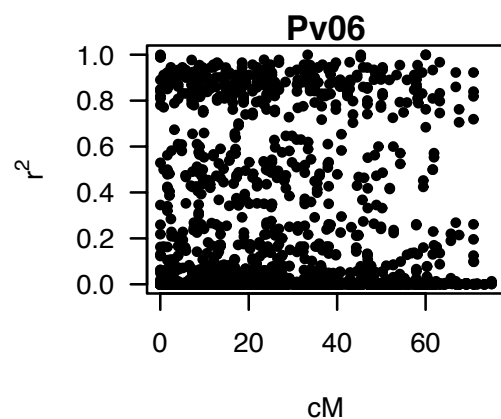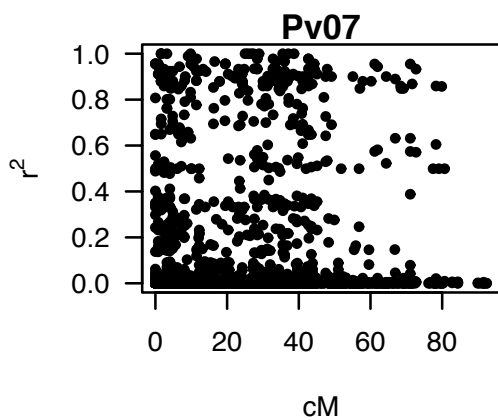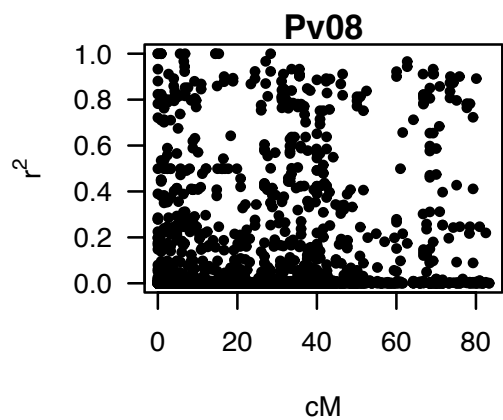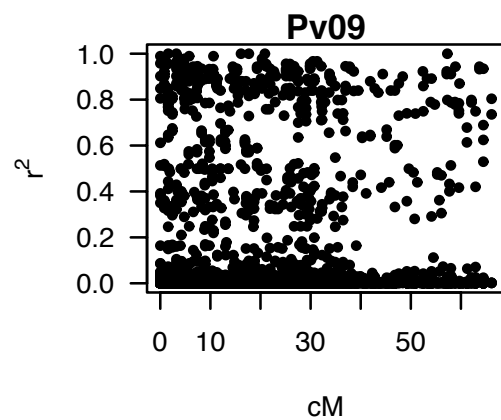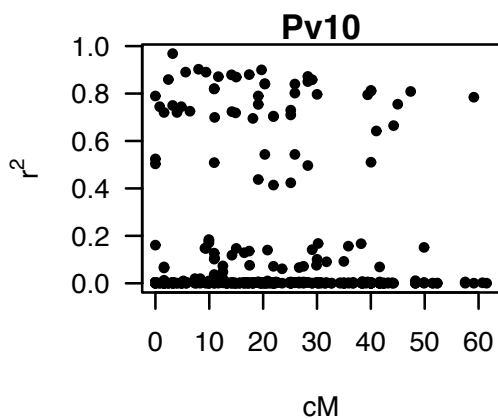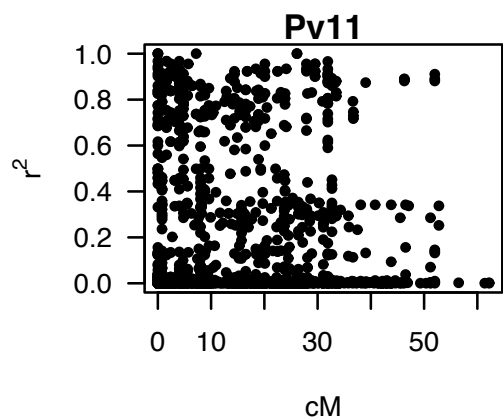

Supplement: S5 Fig — (PDF) [file pone.0189597.s008.pdf]

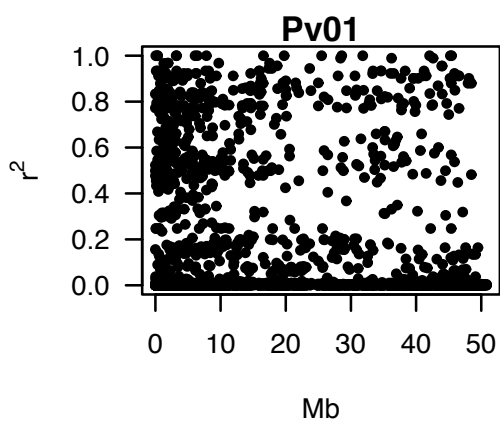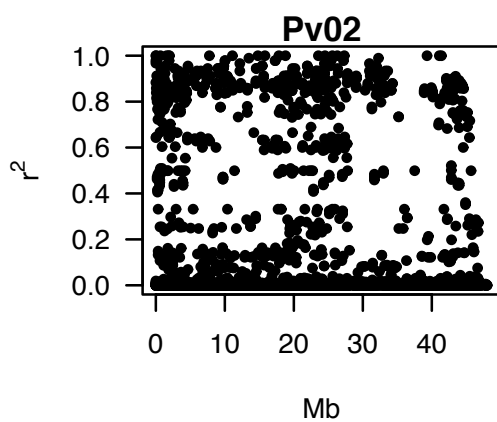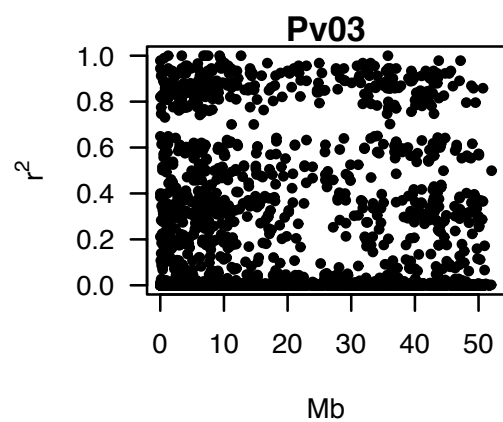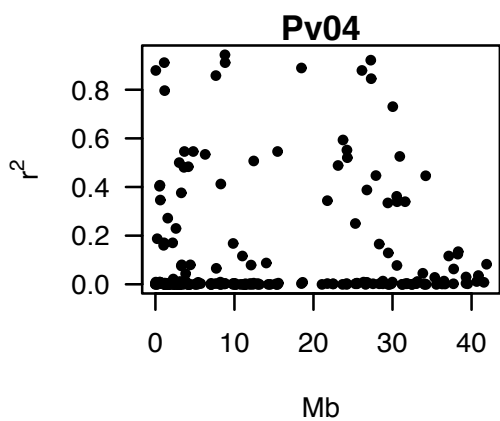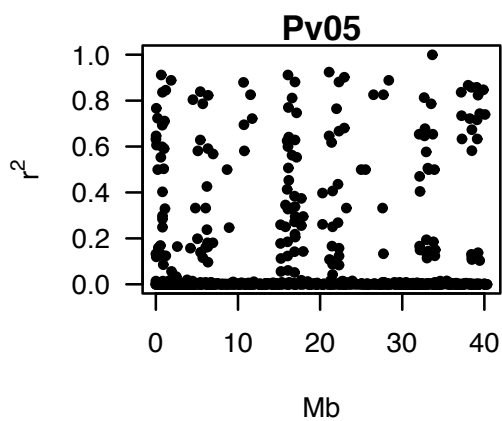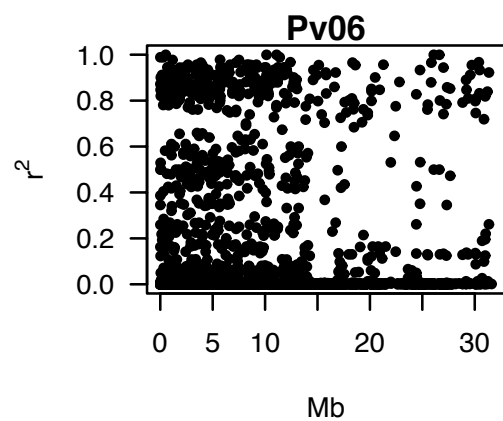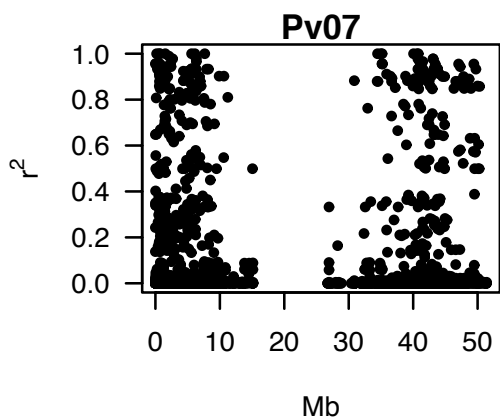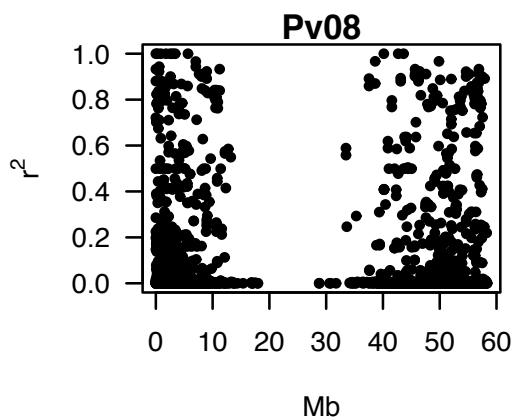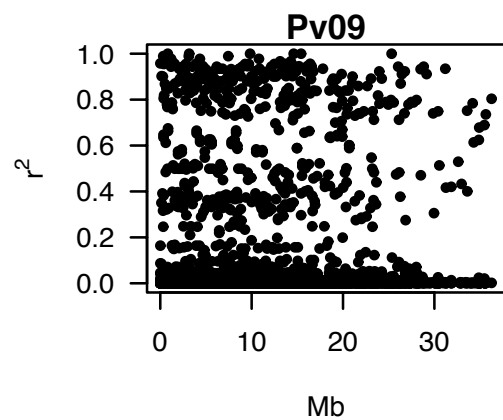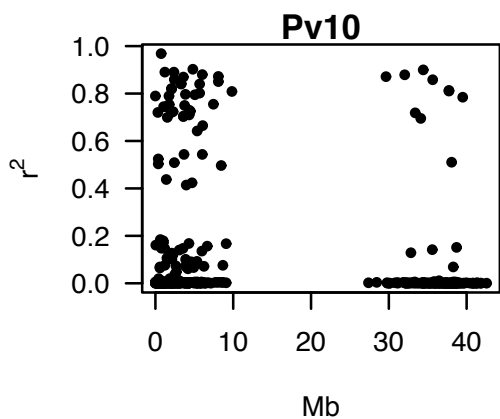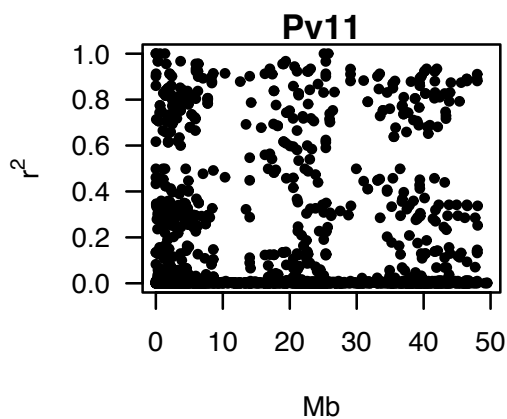

Supplement: S6 Fig — (PDF) [file pone.0189597.s009.pdf]

**Pv01**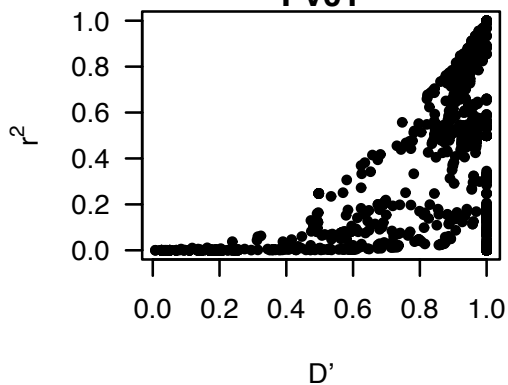**Pv02**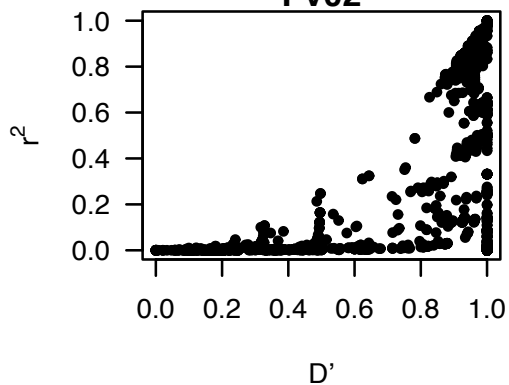**Pv03**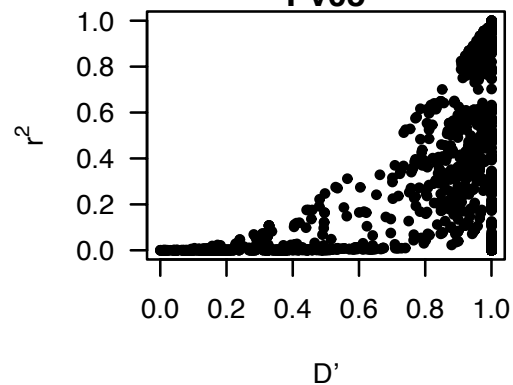**Pv04**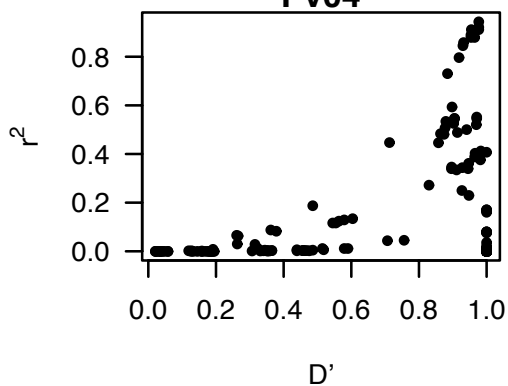**Pv05**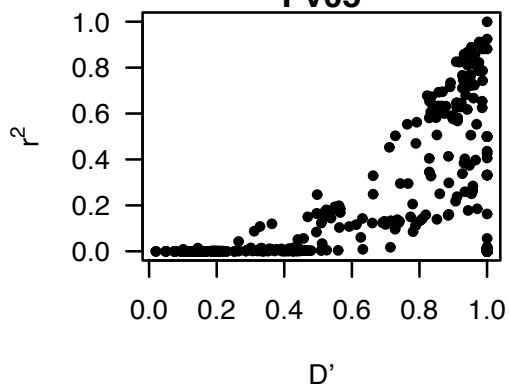**Pv06**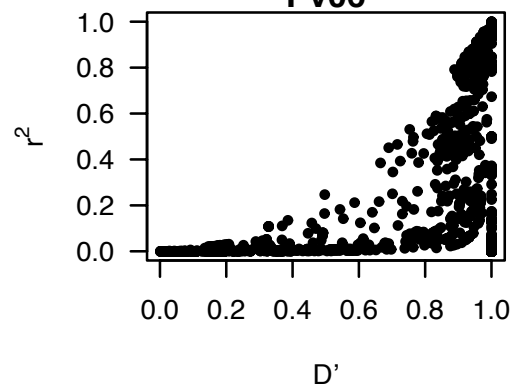**Pv07**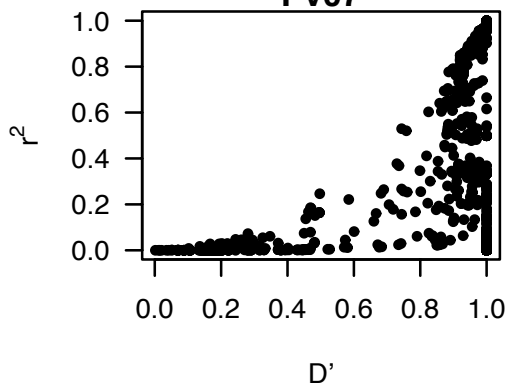**Pv08**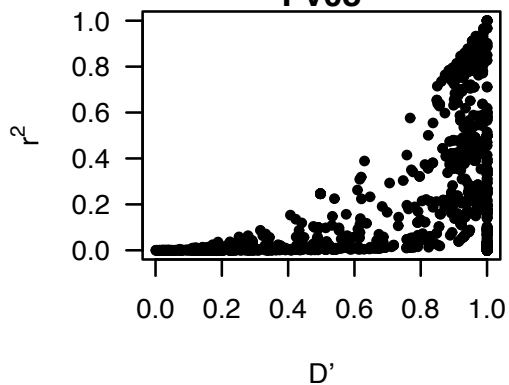**Pv09**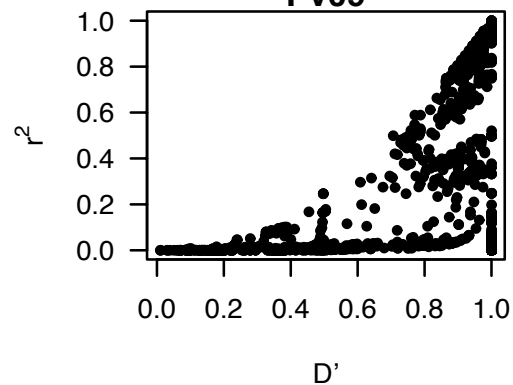**Pv10**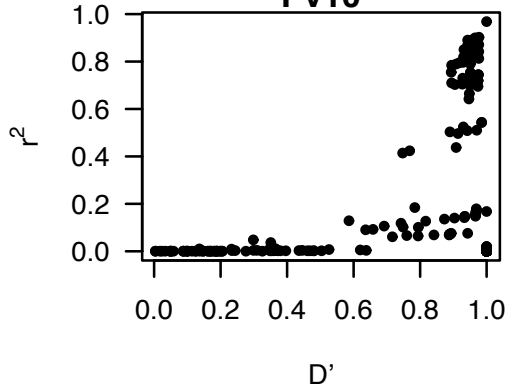**Pv11**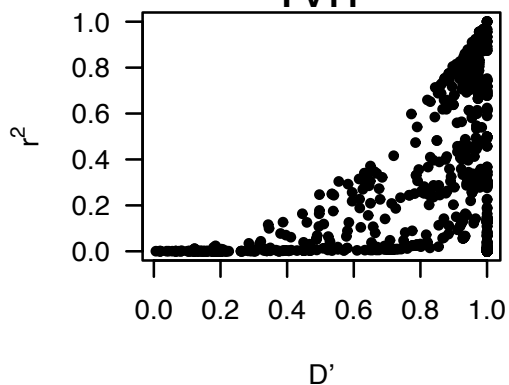

Supplement: S7 Fig — (PDF) [file pone.0189597.s010.pdf]
